# Cannabinoid type 2 receptors in dopamine neurons inhibits psychomotor behaviors, alters anxiety, depression and alcohol preference

**DOI:** 10.1038/s41598-017-17796-y

**Published:** 2017-12-12

**Authors:** Qing-Rong Liu, Ana Canseco-Alba, Hai-Ying Zhang, Patricia Tagliaferro, Monika Chung, Eugene Dennis, Branden Sanabria, Norman Schanz, Joao Carlos Escosteguy-Neto, Hiroki Ishiguro, Zhicheng Lin, Susan Sgro, Claire M. Leonard, Jair Guilherme Santos-Junior, Eliot L. Gardner, Josephine M. Egan, Jeung Woon Lee, Zheng-Xiong Xi, Emmanuel S. Onaivi

**Affiliations:** 1 Department of Biology, William Paterson University, Wayne, New Jersey 74070 USA; 2 Laboratory of Clinical Investigation, national Institute on Aging, National Institutes of Health, Baltimore, MD 21224 USA; 3 Molecular Targets and medications Discovery Branch, Intramural Research Program. National Institute on Drug Abuse, Baltimore, MD 21224 USA; 4 Department of Psychiatry, University of Yamanashi, Yamanashi, Japan; 5 Department of Psychiatry, Harvard Medical School, Psychiatric Neurogenomics, Division of Alcohol and Drug Abuse, and Mailman Neuroscience Research Center, McLean Hospital, Belmont, MA USA; 6 Faculty of Medical Science, Santa Casa, Sao Paulo Brazil

## Abstract

Cannabinoid CB2 receptors (CB2Rs) are expressed in mouse brain dopamine (DA) neurons and are involved in several DA-related disorders. However, the cell type-specific mechanisms are unclear since the CB2R gene knockout mice are constitutive gene knockout. Therefore, we generated *Cnr2*-floxed mice that were crossed with DAT-*Cre* mice, in which *Cre*- recombinase expression is under dopamine transporter gene (DAT) promoter control to ablate *Cnr2* gene in midbrain DA neurons of DAT-*Cnr2* conditional knockout (cKO) mice. Using a novel sensitive RNAscope *in situ* hybridization, we detected CB2R mRNA expression in VTA DA neurons in wildtype and DAT-*Cnr2* cKO heterozygous but not in the homozygous DAT-*Cnr2* cKO mice. Here we report that the deletion of CB2Rs in dopamine neurons enhances motor activities, modulates anxiety and depression-like behaviors and reduces the rewarding properties of alcohol. Our data reveals that CB2Rs are involved in the tetrad assay induced by cannabinoids which had been associated with CB1R agonism. GWAS studies indicates that the CNR2 gene is associated with Parkinson’s disease and substance use disorders. These results suggest that CB2Rs in dopaminergic neurons may play important roles in the modulation of psychomotor behaviors, anxiety, depression, and pain sensation and in the rewarding effects of alcohol and cocaine.

## Introduction

The endocannabinoid system (ECS) consists of genes encoding two major cannabinoid receptors, CB1R and CB2R, endocannabinoids (eCBs), and the enzymes involved in the synthesis and degradation of eCBs^1^. CB1Rs, the most abundant G-protein coupled receptors (GPCRs) in the mammalian brain have been well characterized and conditional *Cnr1* mutant mice^2–4^ have been produced for studies on the role of CB1Rs and have significantly improved our understanding of the mechanism and functional role of CB1Rs.

While the functional neuronal expression of CB2Rs has been a subject of controversy and debate, accumulating evidence^1^ and recent research indicate that neuronal expression of CB2Rs are involved in drug reward and synaptic plasticity^5–7^. Indeed, our previous studies provided the first evidence for neuronal effects of CB2Rs and their possible roles in drug addiction, eating disorders, psychosis, depression, and autism spectrum disorders^1^. However, many features of CB2R gene structure, regulation, function, variants, and impact on behavior remain poorly characterized compared to CB1Rs. CB2R gene knockout mice, two of which are available, contain partial *Cnr2* deletion at C- and N- terminal amino acid sequences and consequently residues of CB2R activities remain^1,8^. These germline knockout mice in which the CB2R function could be compromised by developmental compensation are not suitable for tissue- and cell type-specific studies at molecular, pharmacological, and behavioral levels.

Dopamine (DA) neuron cell type-specific CB2R conditional knockout (cKO) mice are of critical importance in characterizing the molecular basis of CB2R neuronal signaling mechanism and their role in relevant behavioral modifications. Therefore, we generated *Cnr2*-floxed mice that were crossed with DAT-C*re* mice, in which the *Cre* recombinase expression is under DAT-(dopamine transporter) gene promoter control so as to produce *Cnr2*-cKO in DA neurons in DAT-*Cnr2* cKO transgenic mice. This follows the successful production of Syn-CB2R^−/−^ mice in which synaptic deletion of CB2Rs was shown to mediate a cell type-specific plasticity in the hippocampus^7^.

Here we report on the molecular and behavioral characterization, using *in-vivo* and *in-vitro techniques* of the first DAT-*Cnr2* cKO mice to define the specific cell-type functional roles of CB2Rs in DA neurons. Founder mice including the wild type, heterozygous, and homozygous *Cnr2*-floxed as well as DAT-*Cre* mice did not differ in motor function and elevated plus-maze tests. The data obtained from DAT-*Cnr2* cKO mice reveal an inhibitory role of dopaminergic CB2Rs and its deletion in DA neurons modulates psychomotor and rewarding behaviors.

## Results

### Generation of DAT-*Cnr2* cKO mice

**Figure 1:**
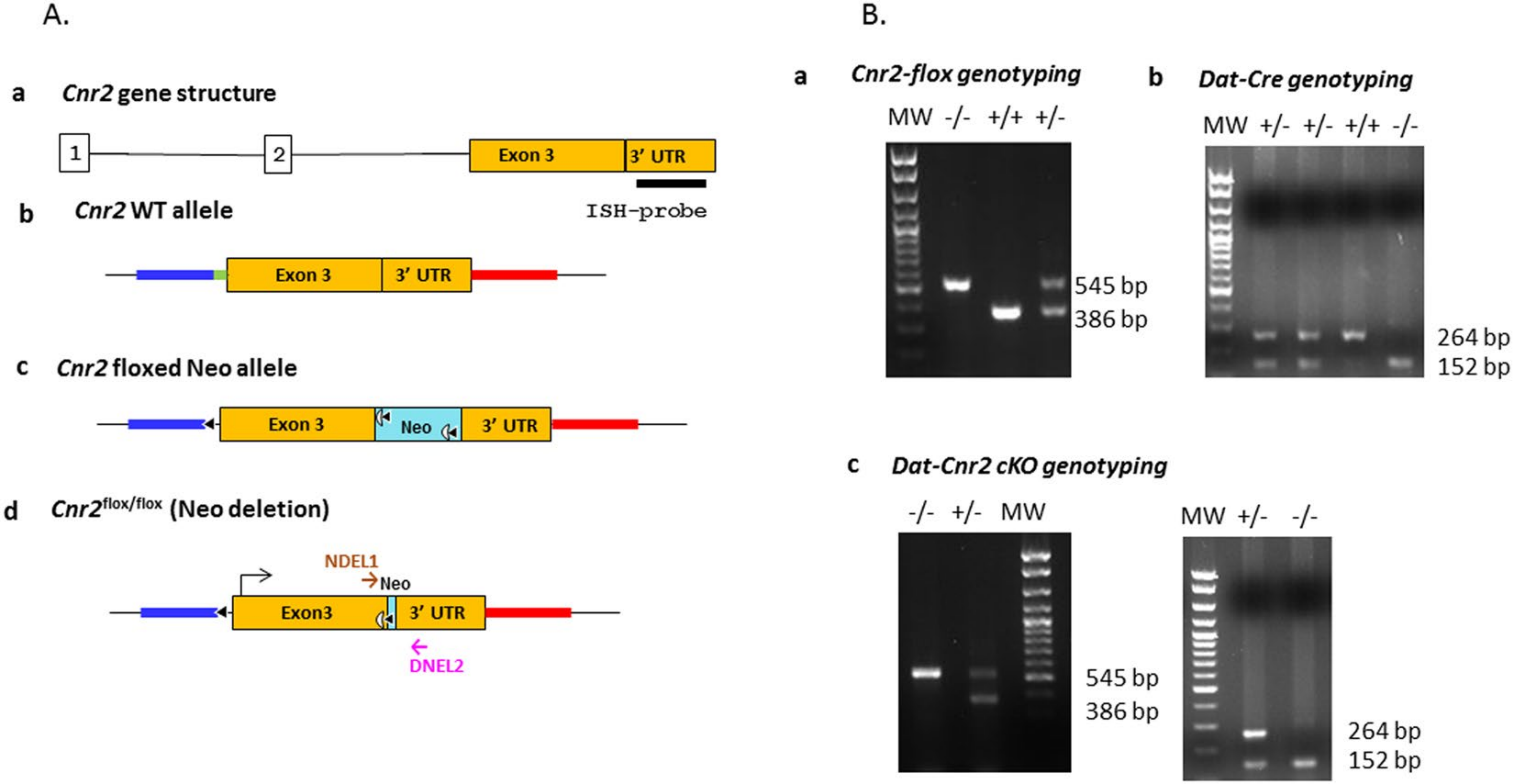
Strategy for the deletion of CB2Rs in DA neurons and genotyping (**A**,**B**). (**A**) Cnr2-loxP targeting strategy: (**A**) **a**, Cnr2 gene structure: exons are marked with open boxes, introns with solid line and black bar RNAscope *in situ* hybridization probe of 897 bp targeting 3′UTR. **b**, the approximate region for the 5′ homology arm (6,060 bp) is marked in red, the approximate region for the 3′ homology arm (3,991 bp) is marked in blue and the splicing acceptor region is marked in green. **c**, the FRT-Neo-FRT/loxP cassette (3′−5′ orientation) is in light blue; **d**, the targeted region including exon 3 and splicing site (2,249 bp) are sandwiched between loxP sites: 5′ loxP is localized upstream (774 bp) of splicing acceptor site of the coding exon 3 and 3′ loxP is localized in the downstream (759 bp) of the stop codon inside 3′ UTR so that the splicing site and the entire open reading frame of mouse *Cnr2* are deleted in dopamine neurons after recombination by mating with dopamine transporter promoter driven *Cre* expressing mouse line (DAT-*Cre*). (**B**) *Cnr2*-flox genotyping: **a**, 386 bp band is a wild type allele and 545 bp band is a mutant allele (MW: molecular weight marker, wt + and mutant -). **b**, DAT-*Cre* genotyping, 264 bp band is a wild type allele and 152 bp band is a mutant allele. **c**, panel shows that DAT-*Cnr2* cKO heterozygous mice are selection of DAT-*Cre* heterozygous genotype and *Cnr2*-flox mutant genotype, DAT-*Cnr2* cKO homozygous mice are the selection of DAT-*Cre* mutant genotype and *Cnr2*-flox mutant genotype.

**Figure 2:**
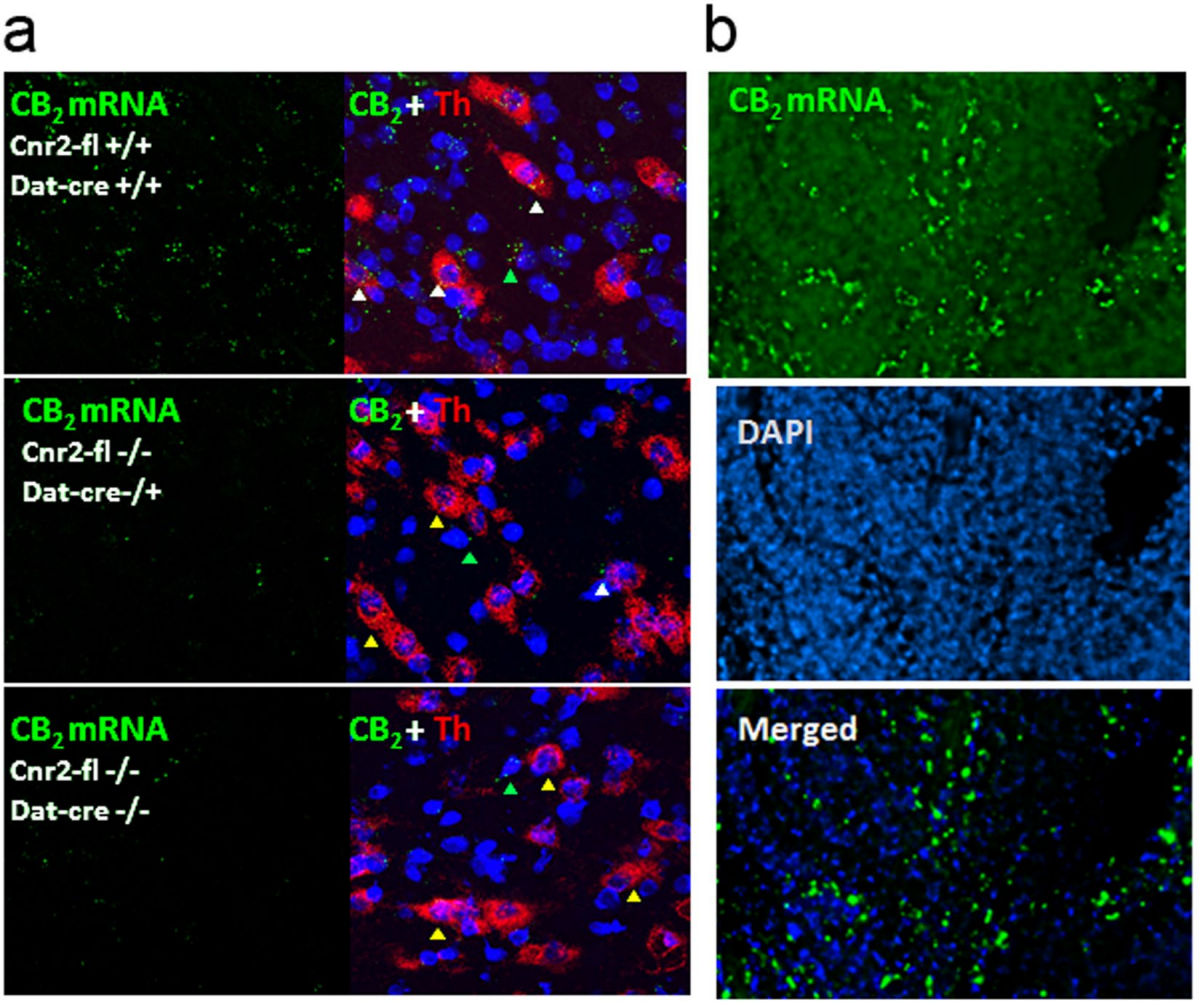
RNAscope *in situ* hybridization using CB2R and tyrosine hydroxylase probes in DAT-*Cnr2* mice (**a**,**b**). (**a**) RNAscope *in situ* hybridization of mouse VTA section, red color represents tyrosine hydroxylase (TH) mRNA, green CB2R mRNA and blue DAPI nucleus staining. Genotypes of *Cnr2*-floxed and DAT-*Cre* are marked with white color on the upper left corners of the sections. White arrow heads represent colocalization of CB2R mRNA and TH mRNA; green arrow heads CB2R mRNA localization in TH negative cell types and orange arrow heads TH positive neurons without CB2R mRNA. (**b**) Spleen section as positive control of CB2R mRNA level that is more abundant in immune cells.

**Figure 3:**
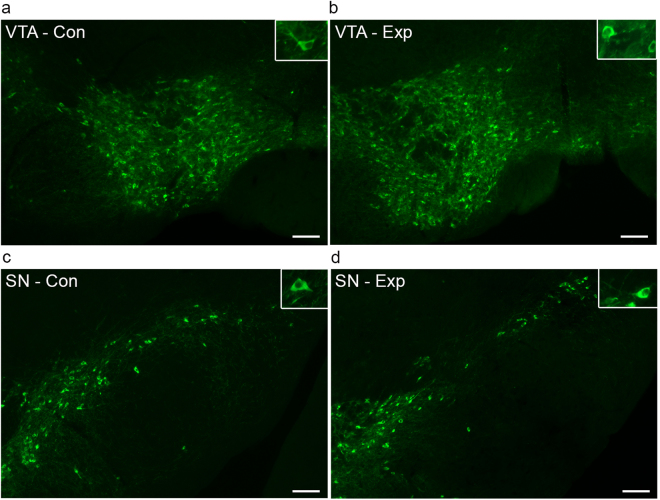
Tyrosine hydroxylase (TH) immunofluorescence staining of the VTA and SN of DAT-*Cnr2* cKO mice after CMS for seven weeks. (**a**–**d**). Tyrosine hydroxylase (TH) immunofluorescence staining in the VTA and SN of non-stressed DAT-*Cnr2* cKO (**a**,**b**) and VTA and SN of stressed DAT-*Cnr2* cKO (**c**,**d**) mice. Microphotographs showing normal anatomical organization and distribution of the total population of dopaminergic neurons of the VTA (**a**) and SN (**b**) of control DAT-*Cnr2* cKO mice and of the VTA (**c**) and SN (**d**) of DAT-*Cnr2* cKO mice exposed to CMS and TH immunofluorescence staining. The morphology and size of individual TH-positive neurons, observed at a higher magnification, also showed a normal appearance in the two studied areas (inserts). Scale bar, 100 µm. This is supportive of the clear detection of CB2Rs in DA neurons in the VTA of wild type and absent in the DAT-*Cnr2* cKO mice in the DA neurons with TH as marker.

In this study we examined the effect of specific deletion of CB2Rs in DA neurons in DAT-*Cnr2* cKO mice. Therefore to determine the role of CB2Rs in this cell type-specific DA neurons, we first generated *Cnr2* floxed mice using Cre-Lox technology. The left loxP site was inserted upstream of 5′- slicing acceptor site of exon 3 and the right loxP at the 3′UTR of exon 3 so that the entire protein coding region of *Cnr2* was deleted upon *Cre* recombination (Fig. 1A). The CB2R floxed mice were crossed with DAT-*Cre* mice, in which the *Cre* recombinase expression is under dopamine transporter (DAT) gene promoter control to generate conditional dopaminergic DAT-*Cnr2* cKO mice. The CB2R floxed, DAT-*Cre* and DAT-*Cnr2* cKO mice were genotyped (Fig. 1B) with primers in (Supplementary Table S3). Duplex *in situ* hybridization (ISH) assay was used to detect neuronal CB2R mRNA expression in the ventral tegmental area (VTA) with *Cnr2* and tyrosine hydroxylase (TH, DA neuronal marker) RNAscope ISH probes (Fig. 2a). The specificity of CB2R ISH probe was validated by positive hybridization signal in monocytes of spleen red pulp (Fig. 2b). CB2 mRNA expression level in brain is ~150x fold less than that of spleen positive controls^9^ and therefore the CB2 mRNA signal in VTA dopaminergic neurons is weak, relative to spleen. However, there was clear detection of CB2R mRNA in DA neurons in the VTA of the wild type and absent in DAT-*Cnr2* cKO homozygous mice providing additional evidence for the dopaminergic expression of CB2Rs in the brain. To determine the integrity of the anatomical organization and distribution of dopamine neurons in VTA and SN in stressed and non-stressed DAT-*Cnr2* cKO and wild type mice, immunofluorescence staining for TH was performed. There was no difference in the overall pattern of TH immunostaining following the deletion of CB2Rs in dopamine neurons. The morphology and size of individual TH-positive neurons, observed at a higher magnification, had a normal appearance in the two areas studied (Fig. 3a–d). For the Western blots in the midbrain, TH protein expression was enhanced in the DAT-*Cnr2* cKO compared to the wild type mice (Supplementary Fig. S2) suggesting an implication of CB2Rs in dopaminergic neurons.

### CB2Rs in DA neurons inhibits motor activities

**Figure 4:**
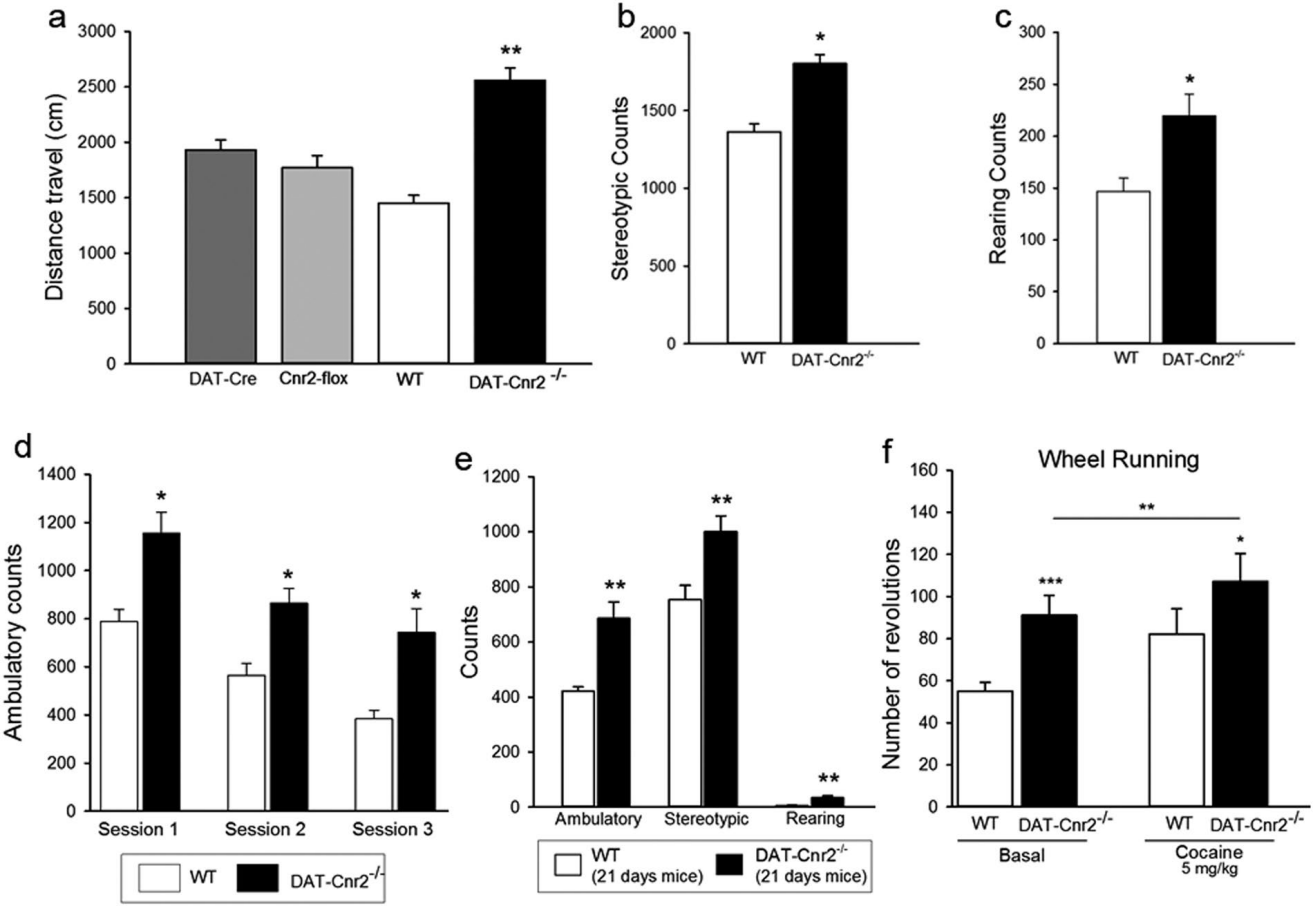
CB2Rs in dopamine neurons inhibits motor activities. (**a**–**f**). (**a**) Establishment of appropriate controls and that DAT-*Cre* and CB2R floxed genotypes do not influence psychomotor behaviors. The locomotor activity measured by the distance travelled were not different in the DAT-*Cre*, *Cnr2*-floxed and wild type mice but activity of the DAT-*Cnr2* cKO mice were significantly (p < 0.01) elevated without drug treatment. (**b**,**c**) There was enhanced stereotype (**b**) and rearing (**c**) behaviors in homozygous DAT-*Cnr2* cKO compared to the wild type mice. (**d**) There was decreased exploratory behavior measured by ambulatory counts in the wild type compared to the DAT-*Cnr2* cKO mice whose ambulatory counts remained elevated. (**e**) By day 21 and throughout adulthood the hyperactivity of the DAT-*Cnr2* cKO mice characterized by increased ambulatory counts, rearing and stereotype behaviors persisted. (**f**) Basal spontaneous wheel running was significantly higher in the DAT-*Cnr2* cKO mice and after cocaine treatment compared to the wild type. Statistical analysis was conducted by two-way ANOVA and data are mean ± SEM, ns P > 0.05, *P < 0.05 or **P < 0.01, comparison of the behavioral effects of DAT- *Cnr2* cKO and wild type mice and after treatment with 5 mg/kg cocaine ip.

To test the hypothesis that CB2 cannabinoid receptors in dopaminergic neurons modulate psychomotor behaviors, we first evaluated the performances of the CB2R-floxed and DAT-*Cre* mice in comparison to their respective wild type C57BL/6 J background mice as controls. As the performances of these groups of mice in the open field and wheel running tests were not significantly different, the C57BL/6 J littermates were used as the wild type controls. The most striking result is the dramatic and significantly elevated motor activity (P < 0.01) of the DAT-*Cnr2* cKO mice (Fig. 4a–f). Next, we evaluated the behavioral characteristics of the DAT-*Cnr2* cKO mice in open field and wheel running tests. Significant and enhanced locomotor activity responses were recorded for the DAT-*Cnr2* cKO compared to both heterozygous cKO and wild type mice in the open field test (P < 0.01). These were characterized by significant increases in locomotor distances covered, stereotypic counts and rearing behavior of the DAT-*Cnr2* cKO mice compared to the wild type mice (Fig. 4a–e). We then tested the occurrence of habituation during three consecutive sessions in the open field test. We found, as expected, that the wild type mice had decreased explorative behavior with familiarity to the task but the DAT-*Cn2* cKO mice, on the other hand, continued to be significantly different (Fig. 4d) compared with the wild type mice. The exaggerated hyperactive locomotor responsiveness by the DAT-*Cnr2* cKO mice was developmentally apparent during weaning with prominent “popcorn” effects by day 21, with persistent and significant ambulatory, stereotypic rearing behaviors (P < 0.01) that were maintained in adulthood (Fig. 4e).

In the spontaneous wheel running test, the wheel running behavior was dependent on the genotype and was significantly higher in the naïve homozygous DAT-*Cnr2* cKO mice compared to those of the wild type mice (Fig. 4f). Furthermore, the DAT-*Cnr2* cKO mice were more responsive to the systemic administration of cocaine (5.0 mg/kg) because it induced significant wheel running behavior compared to wild type mice (Fig. 4f). These results suggest that CB2Rs puts a “brake” on the classical locomotor activation by dopaminergic neuron activation and its deletion in the DAT-*Cnr2* cKO mice enhances mouse psychomotor behavior.

### CB2Rs in DA neurons modifies depression- and anxiety-like behaviors

**Figure 5:**
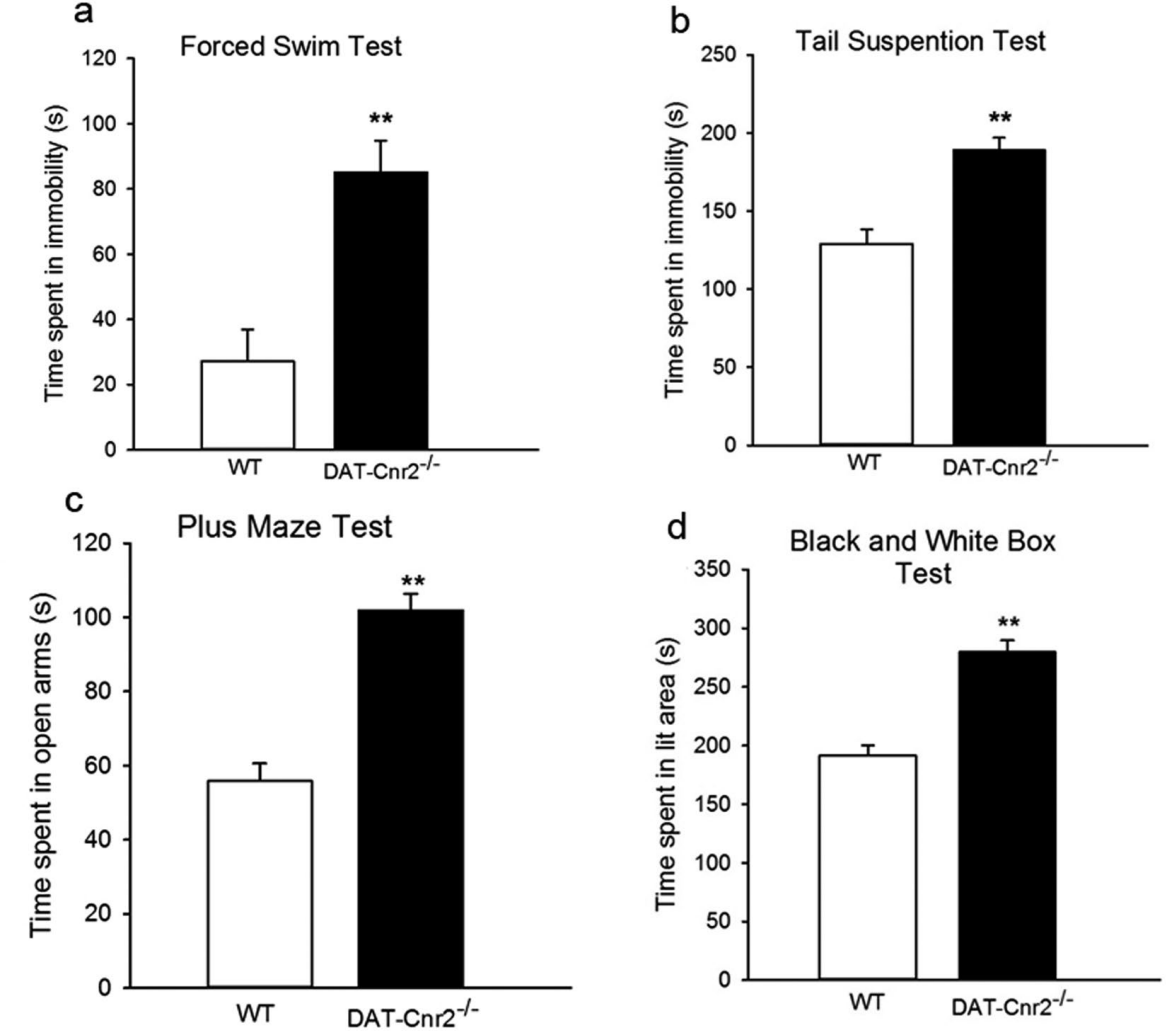
CB2Rs in dopamine neurons modifies depression- and anxiety-like behaviors. (**a**–**d**). In the acute depression-like behavioral tests the DAT-*Cnr2* cKO mice spent significantly more time immobile in the forced swim test (**a**) and in the tail suspension test (**b**) than the wild type mice. Whereas in the elevated plus-maze and two compartment black and white box anxiety tests, the *DAT-Cnr2* cKO mice spent significantly more time in the open arms of the plus-maze (**c**) and the white chamber of the two compartment black and white box (**d**) than the wild type mice. Values are presented as mean ± SEM with 10–12 mice per group and **P < 0.01 for DAT-*Cnr2* cKO and wild type mice.

This previously unknown ECS is emerging as one of the key CNS regulatory systems involved in emotionality nociception, thermoregulation, neurodegenerative, and neuroinflammatory disorders^10^. Because the dopaminergic system is involved in psychosis and neurodegenerative disorders such as Parkinson’s disease, we wondered whether CB2Rs in DA neurons modifies depression- and anxiety-like behaviors that are comorbid with psychiatric disorders. This was accomplished by using measures of depression- and anxiety-like behaviors in the forced swim and tail-suspension tests; two-compartment black and white box and in the elevated plus-maze tests. The results show that in the forced swim and tail-suspension tests, the DAT-*Cnr2* cKO mice spent significantly more time immobile than the wild type mice (Fig. 5a,b: P < 0.01). Curiously, the DAT-*Cnr2* cKO mice were less aversive to the open arms of the plus-maze and the white chamber of the two-compartment black and white box than the wild type mice (Fig. 5c,d). Taken together the data suggest that deletion of CB2Rs in DA neurons plays a role in modulating depression- and anxiety-like behaviors in the anthropomorphic mice emotionality tests.

### CB2Rs in DA neurons and thermo-nociceptive responses

**Figure 6:**
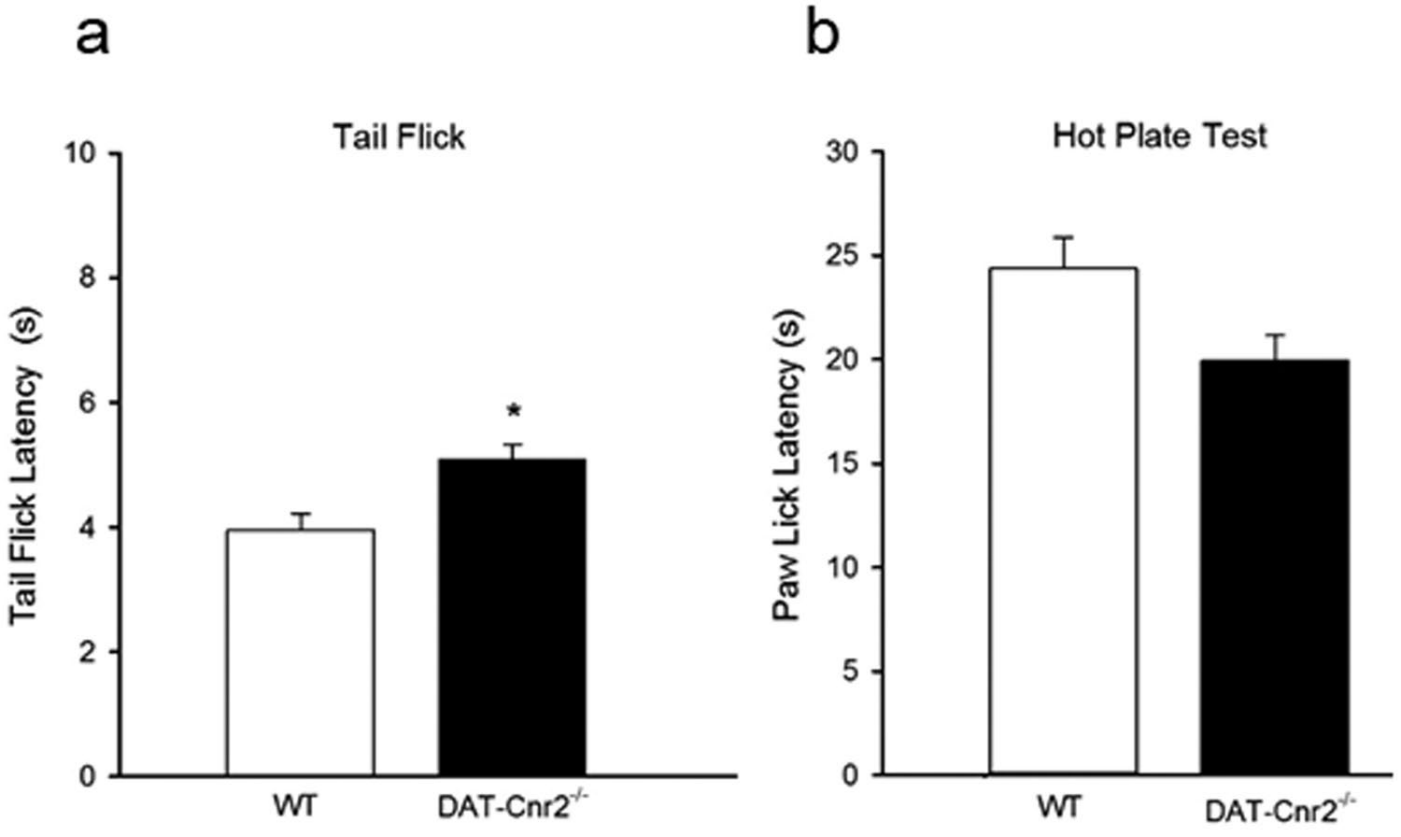
CB2Rs in dopamine neurons and thermo-nociceptive responses. (**a**,**b)**. The tail flick (**a**) latency response was significantly higher P < 0.5 in the DAT-*Cnr2* cKO than in the wild type mice. In the hot plate test (**b**) there was an insignificant decrease ns P > 0.05. Data presented as mean ± SEM with 10 mice per group and *P < 0.05 or ns P > 0.05 for DAT-*Cnr2* cKO and wild type mice in the tail flick and hot plate tests respectively.

CB2Rs have been reported to decrease inflammation^11^ and to play a critical role in cannabinoid-mediated anti-nociception in models of inflammatory pain^12^. The hotplate and tail flick latency tests were used to measure anti-nociception mediated by supra-spinal and spinal mechanism respectively^13^. To assess the role of CB2Rs in DA neurons in thermo-nociceptive responses, the tail flick and hot plate latencies were determined in the DAT-*Cnr2* cKO mice and compared with the wild type mice. We found that the DAT-*Cnr2* cKO mice had significantly higher threshold for the tail flick response along with decreased but insignificant paw lick latency in the hot plate test (Fig. 6a and b: P < 0.05). As nociceptive responses can be measured at different levels, this result suggests that there may be differences mediated by CB2Rs in the tail flick and hot plate tests.

### CB2R mediated behaviors in the tetrad test

**Figure 7:**
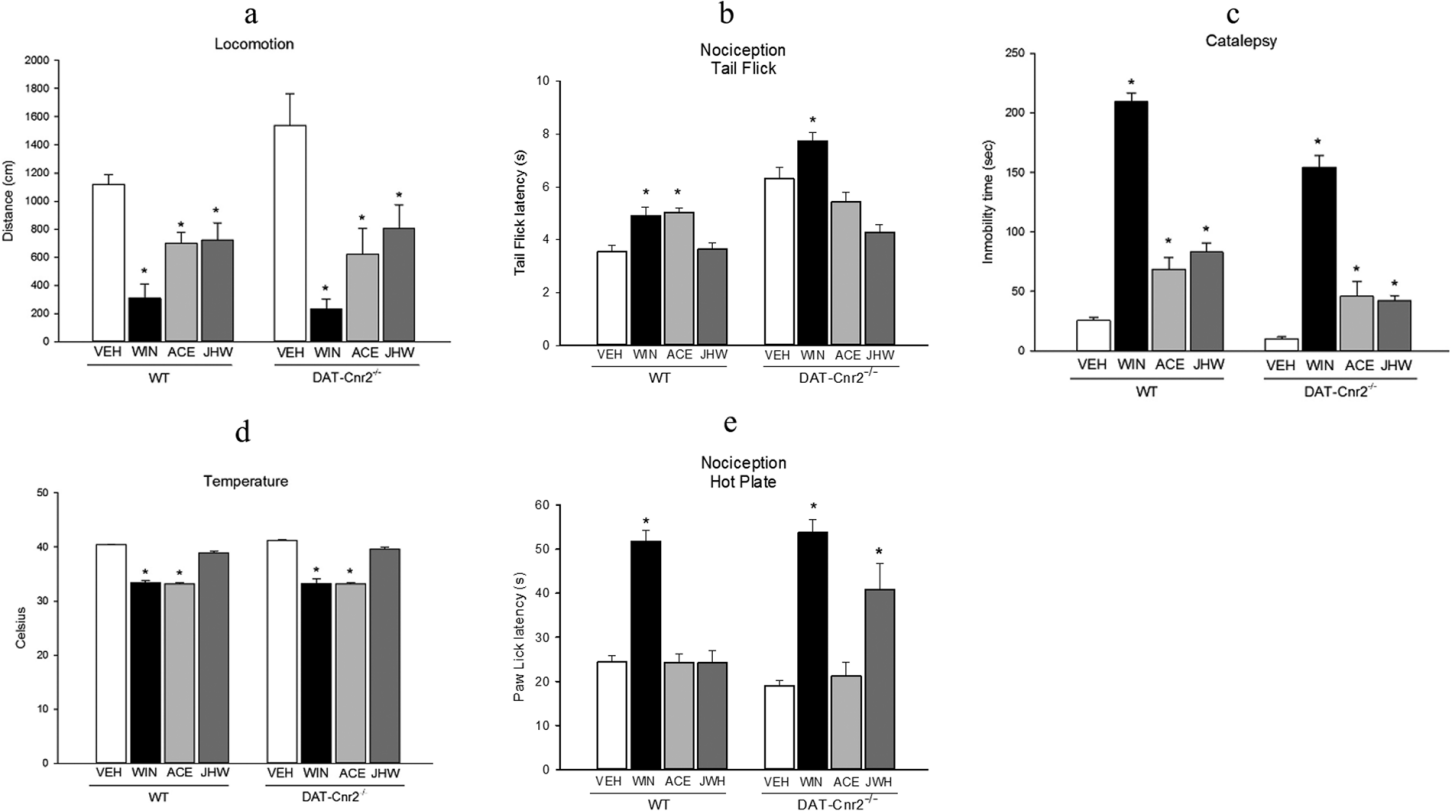
CB2Rs mediated behaviors in the tetrad test. (**a**–**d**). The effects of selected CB1R, CB2R and mixed CB1R and CB2R ligands, WIN 55212-2 (3.0 mg/kg), ACEA (1.0 mg/kg) and JWH133 (20.0 mg/kg) decreased P < 0.05, locomotor activity (**a**) of the DAT-*Cnr2* cKO and wild type mice in comparison to the vehicle treated mice of each genotype. In the tail flick test (**b**) at the doses used, WIN and ACEA but not the JWH compound produced analgesia in the wild type mice whereas in the DAT-*Cnr2* cKO mice only the WIN compound induced analgesia (P < 0.05). In the hot plate test (**e**) WIN was potent in the induction of analgesia in both the wild type and DAT-*Cnr2* cKO mice. ACEA and JWH were ineffective in the induction of analgesia in the wild type mice but JWH and not ACEA produced analgesia in the DAT-*Cnr2* cKO mice. In the catalepsy test (**c**) the treatment with WIN, ACEA and JWH all induced catalepsy (P < 0.05) at the doses used with the DAT-*Cnr2* cKO mice more sensitive than the wild type mice. Rectal temperature (**d**) was reduced by WIN and ACEA (P < 0.05) but not JWH at the selected doses used. Values are presented as mean ± SEM with 10–12 mice per group and *P < 0.05 for DAT-*Cnr2* cKO and wild type mice and their respective vehicle treated controls.

CB2Rs were previously thought to be predominantly expressed in immune cells and their involvement in cannabinoid-induced behaviors was largely unexplored. Traditionally the tetrad effects in mice were used to characterize classical cannabinoids such as Δ^9^-THC that produces the characteristic profile of suppression of locomotion, antinociception, hypothermia and catalepsy which were associated with CB1R agonism. This notion had been supported using data from radioligand binding and *in-vivo* behavioral assays that lacked sensitivity and cell-type specific deletion of the CB2Rs. The dopaminergic neuron-specific deletion of the *Cnr2* gene provided an opportunity to determine the role of CB2Rs in the tetrad tests. We therefore evaluated the effects of selected doses of the mixed CB1R and CB2R agonist WIN 55212-2 (3.0 mg/kg), CB1R agonist ACEA (1.0 mg/kg) and CB2R agonist JWH133 (20.0 mg/kg) to determine whether CB2Rs are involved in the mouse tetrad test using DAT-*Cnr2* cKO mice. Wild type and DAT-*Cnr2* cKO mice were sequentially evaluated in the four tests by measuring locomotor activity, rectal temperature, catalepsy, and anti-nociception after the administration of vehicle or the selected doses of the cannabinoids. The striking result from these studies is the revelation that CB2Rs inhibits motor function and the deletion of CB2Rs in DA neurons induces enhanced motor function characterized by hyper-locomotion of the DAT-*Cnr2* cKO mice (Figs 4 and 7a). Both the wild type and the DAT-*Cnr2* cKO mice were differentially responsive in the tetrad test. In the open field test the mixed CB1R and CB2R agonist, WIN 55212-2 and the selective CB1R and CB2R agonists reduced locomotor activity with the DAT-*Cnr2* cKO more sensitive than the wild type mice (Fig. 7a). In the tail-flick test the systemic administration of WIN 55212-2 and ACEA but not JWH 133 increased the threshold to pain stimulus in the wild type mice whereas in the DAT-*Cnr2* cKO mice only the WIN compound induced analgesia: ACEA and JWH 133 were ineffective in the induction of analgesia at the doses used in the tail flick test (Fig. 7b). In the hot plate test WIN 55212-2 produced analgesia in both the DAT-*Cnr2* cKO and wild type mice. However, ACEA and JWH 133 were ineffective in the induction of analgesia in wild type but JWH 133 and not ACEA induced analgesia in the hot plate test at the doses used (Fig. 7e). In the catalepsy test the systemic administration of the mixed CB1R and CB2R agonist, WIN 55212-2 and the selective CB1R and CB2R agonists all induced catalepsy at the selected doses used with the DAT-*Cnr2* cKO more sensitive than the wild type mice (Fig. 7c). Furthermore, the systemic administration of WIN 55212-2 and ACEA reduced the core rectal temperature whereas JWH 133 at the selected dose that was effective in suppressing locomotor activity in the open field test and inducing catalepsy did not reduce rectal temperature in either the wild type or DAT-*Cnr2* cKO mice (Fig. 7d). Our results suggest that CB1Rs and CB2Rs may contribute to suppression of locomotor activity, antinociception, hypothermia and induction of catalepsy in the cannabinoid tetrad test contrary to long standing notion that the characteristic profile of hypomobility, antinociception, hypothermia and catalepsy were mediated mainly by CB1R agonism^14^.

### CB2Rs in DA neurons modifies alcohol preference and cocaine conditioned place preference

**Figure 8:**
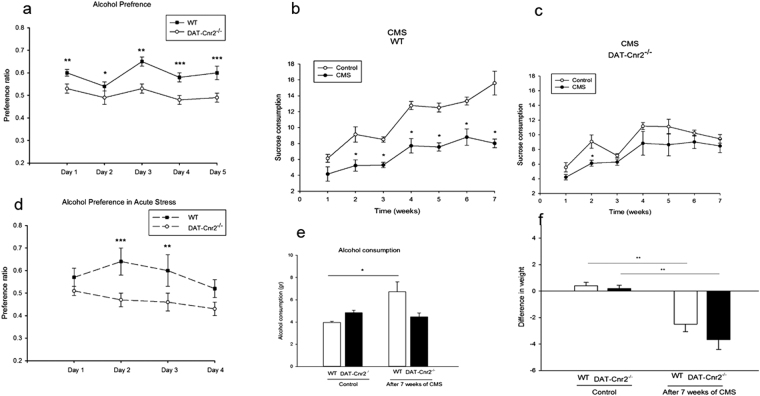
CB2Rs in DA neurons modifies alcohol preference. (**a**–**f**). The 5-day sub-acute stress modifies alcohol preference ratio (**a**,**d**) with the DAT-*Cnr2* cKO consuming less alcohol (P < 0.05) than the wild type mice, with or without acute stressor. In the chronic mild stress model (**b**,**c**), there was significant reduction (P < 0.05) in the consumption of 2% sucrose by the wild type mice in comparison to the DAT-*Cnr2* cKO mice. At the end of the seven week CMS period, there was increased alcohol consumption (**e**) by the wild type mice in comparison to non-stressed wild type and stressed and non-stressed DAT-*Cnr2* cKO mice. There were significant weight changes (**f**) (P < 0.01) following CMS in both the wild type and DAT-*Cnr2* cKO mice compared to non-stressed wild type and DAT-*Cnr2* cKO mice. Data for the CMS studies are presented as mean ± SEM with 6 mice per group and *P < 0.05, **P < 0.01 for DAT-*Cnr2* cKO and wild type mice.

We have previously demonstrated that CB2Rs are involved in alcohol preference in mice and alcoholism in humans^15–18^ and reported on CB2R species differences in responses to cocaine self-administration in mice and rats^5^. Alcohol preference was shown to be enhanced by CB2R agonist in stressed but not in mice that were not subjected to the stress paradigm while CB2R antagonist prevented the development of alcohol preference^16^. The role that CB2Rs in dopamine neurons play in alcohol preference with or without stress was directly examined in DAT-*Cnr2* cKO and the wild type mice using sub-acute and chronic mild stress paradigms. For the sub-acute stress, mice were restrained in a 50 mL conical tube for an hour each day for five consecutive days and the daily consumption of alcohol and water in the two-bottle choice preference test was recorded and alcohol preference ratio determined. Stress modified alcohol consumption regardless of the genotype. However, the DAT-*Cnr2* cKO mice consumed less alcohol than wild type mice with and without the stress (Fig. 8a and d). There was no difference in alcohol consumption between stressed and non-stressed DAT-*Cnr2* cKO mice. Nevertheless, there was significant difference in alcohol consumption between stressed and non-stressed wild type mice in the sub-acute stress paradigm. The alcohol preference ratio was significantly higher in wild type compared to DAT-*Cnr2* cKO mice, suggesting that the deletion of CB2Rs in DA neurons contributed to the reduction in alcohol consumption and preference. The results suggest that the DAT-*Cnr2* cKO mice are resistant to alcohol consumption even during sub-acute stressful condition.

In the next set of studies, wild type and DAT-*Cnr2* cKO mice were subjected to chronic mild stress (CMS) and equal number of the wild type and DAT-*Cnr2* cKO mice were kept in a separate room without CMS for a seven-week period. Anhedonia was assessed weekly by measuring 2% sucrose intake once every week in both stressed and non-stressed wild type and DAT-*Cnr2* cKO mice. In the wild type mice, CMS significantly (p < 0.05) reduced sucrose consumption establishing robust anhedonia whereas in the DAT-*Cnr2* cKO mice there was reduced sucrose consumption by the second week, but after which there were no significant differences in sucrose consumption in stressed and non-stressed DAT-*Cnr2* cKO mice for the remaining test period (Fig. 8b and c), demonstrating an inhibitory role of CB2Rs in hedonic response. At the end of the seven-week CMS experimental period, alcohol intake was not different between the wild type and DAT-*Cnr2* cKO mice. Interestingly the alcohol intake of wild type mice subjected to the seven weeks of CMS were significantly more than the intake of the CMS DAT-*Cnr2* cKO mice (Fig. 8e). In fact, the alcohol intake of CMS DAT-*Cnr2* cKO mice were not different from the wild type and DAT-*Cnr2* cKO mice that were not stressed (Fig. 8e). Another striking result obtained was the significant weight changes induced in the stressed wild type and DAT-*Cnr2* cKO mice compared to the wild type and DAT-*Cnr2* cKO that were not subjected to the CMS (Fig. 8f). The data indicate that CB2Rs in DA neurons may be an important component that is implicated in modifying alcohol preference and stress induced dramatic weight changes. The current findings provide further evidence for the functional neuronal expression of CB2Rs in DA neurons associated with the reward pathway in the CNS.

**Figure 9:**
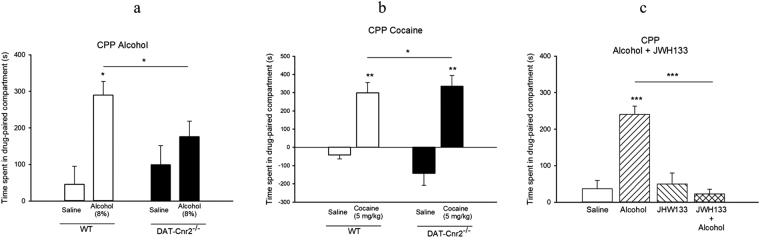
CB2Rs in DA neurons modifies alcohol and cocaine conditioned place preference. (**a**–**c**). Alcohol 8% (**a**) and cocaine 5.0 mg/kg (**b**) induced CPP (P < 0.05) in wild type mice whereas the DAT-*Cnr2* cKO mice did not show significant differences in post-conditioning phase between alcohol, and saline. However cocaine significantly induced CPP in both wild type and was enhanced in DAT-*Cnr2* cKO mice. Alcohol induced CPP (**c**) was significantly inhibited by the selective CB2R agonist JWH133 (5.0 mg/kg) (P < 0.001). Values for the CPP studies are presented as mean ± SEM with 10–12 mice per group and *P < 0.05, **P < 0.01 for DAT-*Cnr2* cKO and wild type mice.

To determine the role of CB2Rs in the rewarding properties of alcohol and cocaine we investigated the effect of CB2R gene deletion in dopaminergic neurons using the conditioned place preference (CPP) of the wild type and DAT-*Cnr2* cKO mice. Wild type mice showed robust conditioning preference for 8% alcohol and 5.0 mg/kg cocaine whereas the DAT-*Cnr2* cKO mice did not show significant differences in post-conditioning phase between alcohol and saline Fig. 9a and b). However, the preference score is significantly higher for cocaine in DAT-*Cnr2* cKO as compared to the wild type control mice. Our previous data showed that systemic administration of JWH 133 dose-dependently inhibited intravenous cocaine self-administration under fixed ratio schedule of reinforcement in mice, but not in rats. Here we also examined the effect of JWH 133 (5.0 mg/kg) on alcohol induced-CPP in the wild type C57BL/6 J mice and found that alcohol induced CPP whereas JWH 133 did not, but JWH 133 reversed alcohol CPP (Fig. 9c). These results imply that DAT-*Cnr2* cKO mice develop less conditioning to alcohol than the wild type mice, suggesting a role for CB2Rs in dopaminergic neurons in alcohol and cocaine preference in the CPP paradigm.

### Human CB2R gene association with substance use disorders and Parkinson’s disease

As dopamine is a major neurotransmitter involved in the regulation of movement, emotions and the feelings of pleasure^19^, GWAS analysis of human CNR2 association with Parkinson’s disease and substance use disorders was evaluated. The results of the meta-analysis of three dbGaP GWAS datasets for CNR2 nominal association with Parkinson’s disease (Supplementary Table S1 and S2) show a signal in 3′UTR, rs4474201 (p = 0.02175, OR = 0.91. These were nominal signals, without passing Bonferroni correction. However, among more than 200 imputation-captured SNPs, we found a positive association cluster around the 3′UTR region of CNR2 gene (Supplementary Fig. S1) for substance use disorder (polysubstance abuse, mainly alcohol and cigarette smoking). The methods and datasets derived from recent study^20^, included in the Supplementary Table S1 and S2 for more related data, supporting Supplementary Fig. S1. The most significant signal came with the SNP rs3123557 (meta-analysis p = 0.002879, OR = 1.15 by random effects). This SNP is a very common variant (C/T), with a minor allele frequency (MAF) of 0.3–0.5 as indicated by the current NCBI database. No positive signals were observed for the 5′ or promoter regions of this gene.

## Discussion

In this study, we report the generation of the first and validated DAT-*Cnr2* cKO mice in which cannabinoid type 2 receptor (CB2R) was deleted from DA neurons and provides a powerful new genetic tool for studying and understanding the functional role of CB2Rs in DA neurons. The major finding is that CB2Rs in DA neurons are involved in motor function, and their deletion releases the “brake” on psychomotor activity so that these DAT-*Cnr2* cKO mice display continuous spontaneous hyperactivity. This exaggerated hyperlocomotor responsiveness was developmentally apparent during weaning with prominent “popcorn” effects characterized by significant ambulatory, stereotypic and rearing behaviors that were maintained in adulthood. TH protein expression was increased in the midbrain of the DAT-*Cnr2* cKO mice compared to the wild type mice supporting a role of CB2Rs in DA neurons. Most strikingly, the deletion of CB2Rs in DA neurons reveals for the first time that CB2Rs are involved in the “tetrad” test which had been previously associated only with CB1R agonism^14^. In addition we demonstrate that CB2Rs in DA neurons play an important role in the modulation of anxiety, depression, and pain sensation and in the rewarding effects of alcohol and cocaine. Furthermore, human CNR2 gene is potentially associated with substance use disorders and Parkinson′s disease. The deletion of CB2Rs in DA neurons reveals an inhibitory role for CB2Rs in CNS function and may underlie the neuropsychiatric potential use of targeting CB2Rs. One of the advances in the neurobiology of CB2Rs was the discovery of functional neuronal expression of CB2Rs^21–23^ and this discovery was followed by the cell-type specific deletion of CB2Rs in synapses of hippocampal CA3 and CA2 pyramidal cells^7^ and by this present report of DA- neuron-specific deletion of CB2Rs to further explore and characterize the neuronal function of CB2Rs.

In DAT-*Cnr2* cKO mice TH immunostaining in VTA area that is involved in psychostimulant drug reward, and in substantia nigra that is associated with psychomotor activities had normal distribution and organization of DA neurons in both stressed and non-stressed DAT-*Cnr2* cKO as well as in wild type mice. This is supportive of the clear detection of CB2Rs in DA neurons in the VTA of wild type and absent in the DAT-*Cnr2* cKO in DA neurons using TH as a marker. In humans, the association signals for CNR2 gene in SUDs represented the first in the field. They were not as strong as they might have been because the underlying variants were not used, and the early GWAS platforms used in the dbGaP studies had relatively low density, thereby making the imputation less effective. However, what’s interesting was the strongest signals came from the 3′UTR of the receptor gene, suggesting that functional variation in the regulatory region of CNR2 confer vulnerability for SUDs. This genetic view is consistent with the preclinical findings in the conditional KO mice, supporting the CNR2 contribution to the etiology of SUDs. DA is involved in the regulation of movement, feelings of pleasure, impulsivity and emotionality. Therefore, disturbances of the dopaminergic system and dysfunction in DA signaling is a major abnormality in psychiatric and neurological disorders^19^. Thus, the current report on the cell type specific deletion of CB2Rs in DA neurons provides a basis for further, and continuing studies in evaluating the role of CB2Rs in the etiology of disorders associated with DA dysregulation.

Functional neuronal expression of CB2Rs has been controversial and a matter of long-standing debate despite new knowledge and advances in the neurobiology of CB2Rs. Furthermore, the mode of action of CB2R agonism, its selectivity and its interaction with CB1Rs have been poorly characterized. This might be due in part to the previous controversial view and ambiguity that the CB2Rs were restricted to peripheral tissues and predominantly in immune cells and was less well studied for CNS function unlike the CB1Rs. Contrary to this view that the CB2Rs were restricted to peripheral tissues and pre-dominantly in immune cells, the current findings provide further additional evidence for the functional neuronal expression of CB2Rs in DA neurons in the VTA - an important reward pathway in the CNS. Since the discovery that the endocannabinoid system is a prominent signaling system and modulator of homeostasis in vertebrates, it has now become a major target of investigation in the mammalian brain for the development of drugs for the treatment of a number of psychiatric and neurodegenerative disorders^24–27^.

In this report our discovery of the inhibition of psychomotor stimulant properties by CB2Rs was confirmed by the genotypic dependent effects of the enhanced locomotor activities of the DAT-*Cnr2* cKO mice providing further evidence for the functional neuronal expression of CB2Rs. Therefore, our data demonstrate the locomotor inhibitory effects of CB2Rs in DA neurons that may play a role in modulating the rewarding properties of psychostimulants and alcohol. However, the role of CB2Rs in tonic endocannabinoid signaling has not been well investigated compared to those of CB1Rs that is associated with the compelling evidence for retrograde synaptic signaling involving 2-AG. This is in part due to many years of CB2R ambiguity and controversy. Nonetheless it has been argued that one of the most recent important advances in synaptic transmission was the discovery that endocannabinoids are the principal mediators of retrograde synaptic communication^28^.

As tonic endocannabinoid signaling is cell type-specific^29^, the selective deletion of CB2Rs in DA neurons provides the opportunity to further examine and characterize the cellular mechanisms underlining the behavioral modifications following deletion of CB2Rs in DA neurons. Although we and others have reported on the functional role of CB2Rs in the CNS^1^, the mode of action is not well established. Our data and those of others^7,21^ sheds new light on the functional neuronal expression of CB2Rs to allow the exploratory therapeutic potential of CB2R ligands. Hence, the results obtained with the deletion of CB2Rs in DA neurons with the striking enhanced spontaneous motor activities can be described as paradigmatic and the underlining associated signaling pathways is in agreement with our demonstration of the postsynaptic and ultrastructural localization of CB2Rs in some brain neurons^30,31^. This is consistent with known CB2R signaling pathways involving Gi/o and the modulation of adenylate cyclase, MAP kinase activity, and IP_3_R activation that results in the opening of Ca^2+^-activated Cl^−^ channels in prefrontal cortical pyramidal neurons that produced a CB2R mediated reduction in firing frequency^21^. The post-synaptic localization of CB2Rs in brain areas where CB1Rs are mostly pre-synaptic makes the interaction between CB1Rs and CB2Rs more complex than previously envisioned. However, this allows both CB1Rs and CB2Rs to work independently and/or cooperatively in differing neuronal populations to regulate differing physiological activities in the brain.

The current studies produced a mouse model with specific behavioral phenotypic characteristic of an overactive spontaneous locomotor drive following the deletion of CB2Rs in DA neurons. Results from the motor function and habituation tests establishes a tonic inhibitory role of CB2Rs in DA neurons and reveals that in the absence of CB2Rs in DA neurons, the “brake” is turned off in the DAT-*Cnr2* cKO mice resulting in the hyper locomotor behaviors. This cell type-specific genetic tool, with the DAT-*Cnr2* cKO mice provided us the first opportunity to examine the role of CB2Rs in the classical cannabinoid tetrad behavioral tests and test the hypothesis that CB2Rs exert cell-type specific neuronal function. Furthermore, the role of CB2Rs in the tetrad test was unknown as the effects of cannabinoids in tetrad assay had been largely associated with CB1R agonism^14,32,33^. Both the CB1Rs and CB2Rs as demonstrated by the effects of the mixed CB1 and CB2 receptor agonist, WIN 55212-2 contributed to the suppression of locomotor activity, hypothermia, analgesia and immobility as expected. Just like WIN 55212-2, JWH 018 that is also a mixed CB1R and CB2R agonist was shown to produce the cannabinoid tetrad responses^32^. But some authors have concluded that the effects of some of the mixed CB1 and CB2R agonists are CB1R dependent effects^32^. Surprising, in this study using mice with dopaminergic neuron-specific deletion of CB2Rs, both the selective CB1R agonist ACEA and CB2R agonist JWH133 differentially contributed to the responses in tetrad tests contrary to the long standing notion that the characteristic profile of hypomobility, antinociception, hypothermia and catalepsy were mediated by the effects of CB1R agonism^14,32^. It should also be noted that the tetrad tests are not entirely pharmacologically specific to cannabinoids^34^, let alone exclusively to CB1Rs as we demonstrated. This study on cannabinoid-induced tetrad assay in mice did not test the possibility that CB2R antagonist will block the effects of CB2R in any of the components of the tetrad tests^33^. Therefore, it appears that both CB1R and CB2R signaling pathways show great diversity and complexity with the unique possibility of similar and different pre- or post- synaptic distribution patterns of CB1Rs and CB2Rs where they may work cooperatively or in opposition to modulate the effects of marijuana, cannabinoid and endocannabinoids in diverse brain areas to maintain homeostasis.

While CB2R post-synaptic localization has been demonstrated in some brain areas^7,30,31^, some unmyelinated axons in the substantia nigra reticulata show CB2R presynaptic localization^30,31^. This agrees with the demonstration that CB2Rs inhibit synaptic transmission albeit in cultured autaptic neurons^35^ and in medial entorhinal area^36^ similar to the effects of CB1Rs whose retrograde signaling function is well established. In the hippocampus and substantia nigra that we have previously demonstrated the postsynaptic localization of CB2Rs, these brain regions are also known to be intensely labeled for CB1Rs and are involved in psychomotor and cognitive function. At a cellular level, it has been suggested that CB1Rs and CB2Rs may provide a non-overlapping functionality with CB1Rs being expressed mostly presynaptically and CB2Rs on postsynaptic compartments of hippocampal CA3/2 pyramidal cells^7^. To our knowledge, our study provides the first direct link between CB2Rs and the classical cannabinoid characteristic profile of effects in the tetrad test. Overall, CB2Rs can be mainly post- or pre- synaptically localized depending on the brain area, just like the CB1Rs that are not exclusively presynaptic, with some postsynaptic distribution reported^37^. Future studies will uncover the molecular mechanisms involved in CB1R and CB2R interaction.

Cannabis and cannabinoids have long been historically used in a number of pain conditions but with limited clinical trials. Cannabinoids appear to modulate and interact with many pain pathways^38^, but the role and mechanism of action of CB2Rs in pain conditions has been less characterized. CB2Rs were previously thought to be predominantly expressed in immune cells in the periphery and were traditionally referred to as peripheral CB2Rs. In contrast to CB2Rs in the periphery, much less is known about the expression of CB2Rs in the CNS and it has remained a subject of debate despite the demonstration of functional expression of CB2Rs in neuronal, glial, and endothelial cells in the brain^1^. It was therefore not surprising that numerous studies have focused on the role of CB2Rs in pathological pain of immune origin both at the spinal and supra-spinal levels using neuropathic and inflammatory pain models. The thermo-nociceptive responses following the deletion of CBR2s in DA neurons in the tail flick test suggests the involvement of CB2Rs in the pain model. This is consistent with elevation in CB2Rs and gene expression in DRG neurons and satellite glial cells in a rat neuropathic pain model^39^. Other preclinical studies using selective CB2R agonists have been shown to attenuate mechanical allodynia and neuroinflammatory responses in rodent pain models suggesting that CB2Rs are intricately involved in the attenuation of inflammatory and neuropathic pain pathways^39–42^. Ongoing studies of the CB2Rs and their possible roles in many pathological conditions including pain of different etiologies may be more complicated than previously appreciated. Nevertheless, a comprehensive analysis of other cell type specific deletion of CB2Rs, particularly in microglial will enhance our understanding of the analgesic mechanism of CB2Rs.

We found that deletion of CB2Rs in DA neurons is implicated in the mouse depression- and anxiety-like behavioral tests which further suggests that CB2Rs in the brain plays a role in modulating emotionality, confirming our previous report that brain neuronal CB2Rs are involved in drug abuse and depression^27^. Accumulating evidence show that there are isoforms of the CB2Rs in human, rat, and mouse with differential subtype distribution patterns in the brain and peripheral organ tissues. The promoter-specific CB2R isoform distribution may in part explain why CB2Rs were previously undetectable in both human and rodent brains^43,44^. The present study found that the DAT-*Cnr2* cKO mice were less aversive to the open arms of the elevated plus-maze and the white chamber of the two-compartment black and white box than wild type mice. On the other hand, in the forced swim and tail suspension tests, the DAT-*Cnr2* cKO mice were more immobile than the wild type mice. This was an intriguing finding and our data show that the DAT-*Cnr2* cKO mice are less anxious in the acute anxiety tests, but more susceptible to depression-like behavior in the acute forced swim and tail suspension tests. Why selective deletion of CB2Rs in dopamine neurons mitigates anxiogenic-like response and induces depressogenic-like behaviors in mice is unknown and warrants further study. However, accumulating evidence demonstrate a complex relationship between CB1Rs and CB2Rs and intracellular signaling in different cell types and pathways. Future studies will determine, the role of CB1Rs following the selective deletion of CB2Rs in DA neurons as CB1Rs agonists are known to produce biphasic dose-dependent effects in rodent models of anxiety. This is consistent with results from the previous pharmacological characterization of cannabinoids in rats and mice using the elevated plus-maze model^45^. In the current study, we found that in the mouse chronic mild stress model, which measures anhedonia - the inability to experience pleasure and one of the core symptoms of depression, the deletion of CB2Rs in DA neurons reduced and blocked the hedonic response to the sucrose consumption and alcohol intake, respectively. This rapidly advancing neurobiology of CB2Rs indicate that CB2Rs are expressed not only in VTA dopamine neurons in mice^5,46,47^, and rats^18^, but also in striatal GABAergic neurons in non-human primates^48^ and in rat VTA astrocytes and microglia^18^. Indeed, CB2R expression in midbrain dopamine neurons in mice and CB2Rs in VTA DA neurons in rats^18^, functionally modulate dopamine neuronal activities is consistent with the current findings presented here.

It is noteworthy that the pharmacological actions of CB1Rs and CB2Rs in the CNS may be more diverse and complex than previously recognized with their differential distribution patterns and species and subtype differences of the CBRs. Moreover, the nature of the interaction between CB1Rs and CB2Rs has not been well characterized. However, the rapidly advancing neurobiology of the endocannabinoid system and emerging evidence suggest that CB1Rs and CB2Rs may work independently and/or cooperatively in different neuronal populations to regulate diverse physiological and biological functions in mental, neurodegenerative, and neuro-inflammatory disorders. In rats it was demonstrated that pharmacological blockade of either CB1Rs or CB2Rs prevented both cocaine-induced conditioned locomotion and cocaine-induced reduction of cell proliferation in the hippocampus of adult male rats^49^. Also, activation of both CB1Rs and CB2Rs was shown to be critical for masculinization of the developing medial amygdala and juvenile social play behavior in rats^50^. Here we confirm a role of CB2Rs in brain DA neurons in the rewarding effects of psychostimulants, alcohol, and cannabinoids in DAT-*Cnr2* cKO mice. This is consistent with the accumulating evidence for the CNS presence and functional role of CB2Rs that modulates DA related behaviors and supports the hypothesis that CB1Rs and CB2Rs may play opposing roles in the regulation of the reinforcing properties of drugs of abuse. Not surprisingly the existence of pharmacological and functional redundancy between endocannabinoid canonical signaling systems in modulation of anxiety-like behaviors is probable, while other non-canonical endocannabinoid inactivation pathways have also been described^51^. Thus, activation of post synaptic localization of CB2Rs in some brain areas^7,30,31^ supports the inhibition of VTA DA neuronal firing by CB2Rs. This may be associated with the resistance of the DAT-*Cnr2* cKO to the induction of conditioned place preference caused by alcohol. However, the DAT-*Cnr2* cKO mice showed an increase to the intensely rewarding effects of cocaine and this contributes to the existing evidence regarding the role of CB2Rs in the reinforcing effects of cocaine. The differential effects of alcohol and cocaine in the DAT-*Cnr2* cKO mice in the CPP paradigm, illustrates the complexity of the role that CB2Rs in dopamine neurons in the effects of drugs of abuse.

The advances and transformation of cannabinoid research into mainstream science may be due, in no small part, to the advancement in molecular biology techniques and the development of highly selective genetic research tools that provides new knowledge and deeper insight in understanding the endocannabinoid system. However there are currently two opposing views on the functional expression of CB2Rs in neurons in the CNS with a minority view that CB2Rs are absent in neurons or have a very limited pattern of expression in the brain. Contrary to this previous prevailing view that CB2Rs are restricted to peripheral tissues and predominantly in immune cells, our data from the analysis DA neuron-specific deletion of CB2Rs and those of others from the analysis of mice with deletion of CB2Rs from synapses demonstrates that CB2Rs mediate a cell type-specific plasticity in the hippocampus^7^ further supports and adds to the mounting evidence of existence and functional neuronal expression of CB2Rs in CNS. In spite of the results from these transformational studies and advances that have been termed a central move for CB2 receptors^7,52^, or hooking CB2 receptor into drug abuse^6,7,53^, there is continuous debate on the functional neuronal expression of CB2Rs in the brain. Our data showed that CB1Rs are not expressed in VTA DA neurons and the major endocannabinoid function in DA neuron in the VTA is mediated by CB2Rs that plays an inhibitory role. One drawback from some previous studies has been the unsuccessful attempts by some groups to generate CB2R cKO mice^54^ and the generation of CB2-GFP with peripheral CB2B promoter driven transgenic reporter mouse line that detected microglial but not neuronal expression of CB2Rs^55^. This report of the initial characterization of the DAT-*Cnr2* cKO mice does not include the determination of neuro-immune connection in the DAT-*Cnr2* cKO and is a limitation in this study because of the preponderance of evidence from previous and current knowledge of the importance of CB2Rs in immuno-cannabinoid activity. Certainly, the generation of mouse microglial cell type-specific deletion of *Cnr2* gene will shed light on CNS immune-cannabinoid activity. We also did not determine whether the use of CB1R and/or CB2R specific antagonists will block CB2R mediated tetrad effects reported here as future studies will determine the contribution of CB1Rs and CB2Rs on cannabinoid neurobehavioral alterations and neuro-immune cross talk in the mouse model. Further extensive DAT-*Cnr2* cKO mice electrophysiological characterization will be required to gain a better understanding of the role and mechanism of CB2R function in the nervous system.

In conclusion, we report on the first successful generation of dopaminergic neuron-specific deletion of *Cnr2* gene to produce DAT-*Cnr2* cKO mice and provide evidence for the functional neuronal expression of CB2Rs. Our data also reveal for the first time that CB2Rs are involved in the tetrad assay induced by cannabinoids that had been largely associated with CB1R agonism. The results obtained suggest that CB2Rs in DA neurons may play important roles in the modulation of psychomotor behaviors, anxiety, depression, and pain sensation and in the rewarding effects of alcohol and cocaine. Thus, we have established and generated DAT-*Cnr2* cKO mice that is a useful animal model to study the role of CB2Rs in DA neurons. Our findings reveal CB2Rs as potential new targets of investigation for possible therapeutic development in neuropsychiatric and neurological disorders as the human genome wide association studies (GWAS) secondary analysis indicate that the *CNR2* gene is associated with Parkinson’s disease and substance use disorders.

## Materials and Methods

### Reagents

Cannabinoid ligands, the mixed CB1R and CB2R agonist, WIN55212-2, and the selective CB2R agonist were obtained from Cayman Chemical Company and the selective CB1R agonists, arachidonyl-2′-chloroethylamide (ACEA) was obtained from Tocris Bioscience. Alcohol as absolute ethanol from Pharmaco-AAper, Bristol, PA and cocaine from Sigma, St. Louis, MO. The primers used for genotyping and RNAscope *insitu* hybridization are provided in (Supplementary Table S3).

### Drug treatment protocol

Cannabinoid ligands, the mixed CB1R and CB2R agonist, WIN55212-2, and the selective CB2R agonist JWH 133 were made up in tween 80: DMSO: Saline in a ratio of 1:2:7. The vehicle was the same ratio 1:2:7, of tween 80: DMSO: Saline. ACEA, the CB1R selective agonist was purchased already made up in Tocrisolve-100 and made up to the selected dose using the vehicle. The doses of cannabinoids ligands, alcohol 8–16% and cocaine (5 mg/kg) dissolved in normal saline were based on our previous research^6,16^. The route of administration of the drugs were dependent on the behavioral test, with alcohol consumption in the stress models and injection in the conditioned place preference paradigm as described below. All drugs were injected intraperitoneally (IP) in a volume of 10 ml/kg body weight.

### Generation of CB2^flox/flox^ experimental animals

In collaboration with inGenious Targeting Laboratory (iTL, Ronkonkoma NY) the Cre-LoxP constructs were designed and the CB2^flox/flox^ mice generated at iTL and breeding pairs shipped to our animal laboratory facility at William Paterson University. The strategy used to create the CB2^flox/flox^ mice involved microinjection of targeted iTL BA1 (129 Sv x C57BL/6) hybrid embryonic cells into CB57BL/6) blastocysts. The resulting chimeras with high percentage agouti coat color were mated to C57BL/6 FLP mice to remove the Neo cassette. The coding exon of *Cnr2* are flanked by left LoxP at 5′-splicing site and right LoxP at downstream of the stop codon so that *Cre-* recombination produced the cell-type specific deletion of entire coding region and splicing site. The CB2^flox/flox^ mice were bred and crossed with DAT-*Cre* mice to produce DAT-*Cnr2*-LoxP transgenic mice – making this the first reported cell-type selected deletion of CB2Rs in dopamine neurons. Animals were housed in temperature and humidity controlled animal laboratory with water and food available *ad libitum*. Experiments were performed using adult mice 20–30 g body weight with N = 10 ± 2 mice each for the different experimental groups for the behavioral analysis. The experimental procedures followed the Guide for the Care and Use of Laboratory Animals and were approved by William Paterson University animal care and use committee.

### RNAscope *In Situ* Hybridization (ISH)

RNAscope ISH was performed as previously described^5,18^. RNAscope *in situ* hybridization of mouse spleen section was first performed to demonstrate CB2 probe specificity, green color represents CB2R mRNA, TH and DAT red, and DAPI blue and color joined using iVision software^46^. The animals (DAT^−/−^ *Cnr2* ^−/−^, DAT^+/–^ *Cnr2* ^−/−^, DAT^+/+^- *Cnr2* ^+/+^; 3 mice aged 2–3 months of each genotype) were anesthetized, perfused with saline solution to wash out blood cells in the brain. The mice were then perfused with 4% paraformaldehyde. The brains were removed and postfixed with the same 4% paraformaldehyde solution overnight at 4 °C. The brains were cryoprotected in 30% sucrose in 0.1 M ophosphate buffer overnight, after which the brains were wrapped in labeled aluminum foil for storage at −80 °C for RNAscope ISH. Briefly, 12 µm coronal brain sections were cut and the fixation, protease pretreatment, probe hybridization, preamplification and fluorescent labeling steps were carried out according to the user manual. Wide-field fluorescent images of the ventral tegmental area was captured using a QimagingExi Aqua Camera (Biovision) attached to a Zeiss AXIO Imager M2 microscope using a × 40 objective (Zeiss PLAN-APOCHROMAT, NA = 1.3) with oil immersion. Images were deconvoluted with Huygens software (v3.7, Scientific Volume Imaging). Image Processing and Analysis by Java (ImageJ, NIH) software was used to quantify mRNA signals in the sections.

### Immunohistochemistry

Stressed and non-stressed DAT-*Cnr2* cKO and wild type mice were prepared for immunohistochemical procedures as previously described^56^ and were optimized for tyrosine hydroxylase immunostaining for dopaminergic neurons at the level of the ventral tegmental area (VTA) and substantia nigra (SN). Animals were anesthetized with a ketamine/xylazine solution and perfused intracardially with 0.9% NaCl followed by 4.0% paraformaldehyde (PF) in 0.1 M phosphate buffer (PB), pH 7.1. Brains are removed, post-fixed in the same fixative solution overnight and cryoprotected in 20% sucrose in 4% PF in PB and then frozen. Serial coronal cryostat sections of the brainstem containing the SN and VTA areas were cut at 30 µm. The sections were processed free floating in blocking buffer (0.5% bovine serum albumin in PB) for 1.0 hr at room temperature, and then sections were incubated with the primary antibody rabbit anti-TH (Millipore, Billerica, MA) at 1: 750 for 48 hours at 4 °C. Sections were washed to remove the primary antibody and incubated with the secondary antibody for 1.5 hr at room temperature. Sections were rinsed, mounted onto slides and cover slipped. The sections were analyzed under an Olympus BX51 microscope equipped with a camera connected to a PC. The images were acquired with Cell-F software.

### Behavioral analysis

The successful creation and production of mice with cell specific deletion of CB2Rs in dopamine neurons allowed us to analyze and characterize the DAT-*Cnr2* cKO mice in models of CNS function. For motor function tests of general activity, we used the open field and wheel running tests. For mouse models of emotionality tests we used tail suspension (TST) and forced swim test (FST) to evaluate depression-like behavior while the elevated plus maze and two-chamber light/dark box were used to evaluate anxiety-like behavior. The role that CB2Rs in dopamine neurons play in alcohol preference and cocaine reward using the conditioned place preference following sub-acute and chronic mild stress(CMS) was determined. Anhedonia was evluated weekly using the sucrose preference test. The tail flick and hotplate tests were used to measure the thermo-nociceptive responses. The availability of these mice is an excellent opportunity to reveal whether or not CB2Rs are involved the characteristic cannabinoid induced profile in the “tetrad” test. Animals were allowed to habituate in the test area for at least 30 min before each test. The specific experimental protocols to characterize the involvement of CB2Rs in dopamine neurons in behavioral studies are described below.

### Motor function tests

The open field test was used to assess the general activity, including ambulatory, stereotypic, rearing and jump counts of the DAT-*Cnr2* cKO mice and wild type controls. For this test animals were placed in individual test boxes connected to a computer for 10 mins and activity counts were obtained and analyzed. Open field test is useful for the assessment of general motor function tests, as repeated exposure to the test provides a method for assessing habituation to the increasingly familiar chamber enviroment. To test if habituation developed, the wild type and DAT-*Cnr2* cKO mice were allowed to freely explore the open field chambers for 10 mins for three consective days at the same time. The activities were analyzed and compared between the two groups. Wheel running activity of the animals were monitored by the placement of each mouse in the spontaneous wheel running monitor. The wheel running behavior of the animals were monitored by auto-counters which monitor the total number of revolutions over the 10 mins test session for each animal.

### Emotionality tests

Forced swim test (FST) and tail suspension test (TST) were used to evaluate depression-like while anxiety-like behaviors were assesed using the elevated plus-maze and the two compartment black/white box as we previously utilized^57^ and validated^58^. These tests were used because they have differing sensitivities for predicting depression-like and anxiety-like profiles in the mouse models described below:

#### Forced swim and tail suspension tests

For the FST, mice were first conditioned to water-filled glass cylinders for 20 mins the day prior to testing. The glass cylinder is (16 cm diameter and height 35 cm) filled to a depth 15 cm with water (23–25 °C). One glass cylinder was used for each mouse and we tested six mice at a time using six glass cylinders. In this study a two-day swim test procedure was utilized first to access the basal performance of the different mouse strains. On the first day mice were placed in the glass cylinder with water to the specified depth, and all animals were exposed for 20-min pre-swim test prior to the 5-min forced swim test on day 2. Fresh water was introduced prior to each test. The data recorded during the 5-min test session were the times the animals were immobile and also the number of immobility counts during the test session. The immobility time and counts were retrieved from the recorded test sessions. Similar data was obtained for the vehicle treated control animals. During the test session the duration of immobility was defined by the animal’s stationary position, and only made the minimal movements necessary to keep their head above water.

The tail suspension test (TST) was used to evaluate depression-like behavior in the mice. Each mouse was suspended by the tail so that the body dangles in the air, facing downward from a metal bar elevated 30 cm. The behavioral profile in this test was videotaped for 6 min and immobility times during the tail suspension was scored using stopwatch from the videotape and analyzed. The tail suspension test was performed as previously described^59^.

#### Two compartment black/white box and elevated plus maze test

An insert with black and white compartment and inter-connecting opening was inserted into each of the locomotor activity boxes (a total of 5 boxes and 5 inserts) and used to measure anxiety-like behavior in the test subjects. Animals were habituated to the test environment before placement individually into the center of the white area and their behavior and movement was recorded with the computer interphase. The time spent and number of entries into the compartments were retrieved and analyzed for DAT-*Cn2* and wild type mice. The elevated plus maze (EPM) was used as a screening test for anxiety-like behavior and data was compared to that obtained from the black/white box. The EPM test setting consisted of a plus-shaped arena with two open and closed arms, each with an open roof, elevated 60 cm from the floor. The test was initiated by placing each mouse on the central platform of the maze, facing one of the open arms, and letting it move freely. Each session lasted for 5 min. Six mice per group were used and their respective times spent in the open and closed arms, as well as the number of entries into the arms, were recorded. The maze was cleaned with alcohol between tests. This procedure is similar to that previously used for the pharmacological characterization of cannabinoids in the plus maze^60^.

### Thermo-nociceptive tests

Hot plate and tail-flick tests were used to evaluate the responsiveness to thermal stimulus. Pain responses from these tests constitute part of the characteristic profile of cannabinoid effects in the tetrad tests described below and after the administration of cannabinoids. For the hotplate test, animals were gently dropped into a box with a metal floor that was pre-heated to 55 ± 2 °C. The latency to responses such as jumping, licking a hind paw or flinching one of the paws is taken as a nociceptive response. To prevent tissue damage, a cut off time was set to 90 sec. The tail-flick was included to assess the latency of the avoidance to thermal stimulus in the mice and also after the administration of cannabinoids in the tetrad tests. In this test radiant heat was applied to about 4 cm from the tip tail length using a tail flick apparatus (UGO Basil). Tail-flick latency time was measured as the time from the onset of the heat exposure to the time of withdrawal of the tail, when the animal feels discomfort, and reacts by a sudden tail movement – the tail-flick response. A cut-off point of 15 s for the discontinuation of the heat stimulus was used to avoid tissue damage. For each mouse tested, baseline latency was obtained as the mean of three measurements.

### Tetrad tests

The characteristic profile of suppression of spontaneous locomotor activity, anti-nociception, hypothermia and catalepsy which are referred to as the tetrad tests, has been associated with the effects of classical cannabinoids and postulated to be mediated by CB1R agonism. We hypothesized that CB2Rs are involved in the tetrad test. To test this hypothesis, we first evaluated the performances of the DAT-*Cnr2* cKO and the wild type mice in the tetrad tests, and then determined the effects of selected cannabinoids in these animals in the tetrad tests. Briefly, the mouse tetrad tests consist of four simple evaluations, which were measured in sequence as we previously described^61^. On the test day, DAT-*Cnr2* cKO and wild type control mice (N = 10 ± 2 animals per group) were evaluated in the tetrad test by the measurement of: 1) activities in the locomotor activity boxes for 10 min, 2) catalepsy, amount of time in 5 mins that the animal remains immobile in the ring test, 3) rectal temperature and 4) nociception, measured by the hot plate and tail flick response. The experiment was repeated following intraperitoneal (i.p) administration of selected doses of the mixed CB1R and CB2R WIN 55212-2 (3.0 mg/kg), CB1R agonist (ACEA (1.0 mg/kg) and CB2R agonist JWH 133 (20.0 mg/kg).

### Alcohol preference test

All tests of preference were conducted in individually housed DAT-*Cnr2* cKO and the wild type mice (N = 12 mice per group) with two fluid bottles available to the animals, for a 24 h period. To establish a baseline of consumption both bottles were filled with 150 ml of water and weighed with the tops and placed over the cages for three days. For the preference measurement one bottle was replaced with 16% alcohol. The bottles were weighed for each animal for five consecutive days at 10 am. The positions of the bottles in the different cages were randomized with respect to which side of the cages they were placed. In all experiments, the ratio of alcohol to water consumed, and the total fluid consumption, were calculated to obtain a preference ratio. Half of the animals in each group (N = 6) were stressed by putting them in a 50 mL tube for an hour each day for 5 consecutive days. Alcohol preference ratio was determined by dividing the amount of alcohol consumed by total fluid (alcohol + water) consumed with and without the sub-acute stress.

### Conditioned place preference

Alcohol and cocaine CPP training and testing were conducted using an infrared photobeam detector open field apparatus (ENV-510) from Med Associates (St. Albans, VT, USA) equipped with the two-compartment place preference inserts (ENV-512). The floor for chamber-1 has parallel rods (3-mm radius, 8 mm center to center spacing) with black cardboard paper covering the outside. Chamber-2 has a stainless steel wire mesh (6 × 6) floor with white cardboard paper covering the outside of the walls. Each compartment is 13 cm (width) × 24 cm (length) × 15 cm (depth). An Activity Monitor software (Med Associates, St. Albans, VT) was used for automated data collection. All conditioning sessions and preference tests were performed between 10 am and 1 pm., with each group of DAT-*Cnr2* cKO and control mice tested at the same time every day. For each group the experiment took place over a period of 8 days split into three phases (pre-conditioning, conditioning and post-conditioning). Each phase is separated by one day. In the pre-conditioning phase, before the first conditioning session, mice were placed in the room with the CPP apparatus for approximately 45 minutes for them to habituate. After habituation, subjects were placed in CPP box and allowed 15 minutes of unrestricted access between sides, the time spent in each of the compartments was recorded using Activity Monitor®. During the conditioning phase animals were injected with saline by their weight and confined to compartment 2 of the CPP apparatus for 15 minutes. Four hours after the first session, mice designated as control received ip vehicle injections once more and placed in compartment 1 for 15 minutes. The other half of the animals received alcohol or cocaine by their weight and also confined to compartment 1 for 15 minutes. This was repeated for 4 consecutive days. At the end of each session, mice were immediately removed from the CPP compartments and returned to their home cages. On the test session (day 8), or post-conditioning phase, mice were allowed to freely explore both sides of the CPP compartments for 15 minutes (identical to day 1). The activity monitor recorded time spent in each of the compartments. The time which each mouse spent in the compartments was recorded and the CPP score was defined as the time spent in the drug paired compartment minus the time spent in the saline-paired compartment during the CPP test.

### Chronic mild stress and anhedonia test

Two groups of DAT-*Cnr2* cKO and WT mice (N = 6 per group) were subjected to CMS for a period of 7 weeks. The other two separate groups of DAT-*Cnr2* cKO and WT mice (N = 6 per group) were the experimental control groups that were housed in a separate holding room and were not subjected to CMS. All animals were housed individually in their cages for the duration of the study. The CMS procedure was performed according to the method we used previously^62^, with some modification. Briefly, mice were subjected to various stressors according to a semi-random schedule for 7 consecutive weeks. The stress regime for each week consisted of food deprivation for 12 h, water deprivation for 12 h, damp bedding for 12 h, overnight stroboscopic illumination, tail suspension for 10 min, tube restrained stress for 30 min, loud music overnight, wire mesh to replace bedding, 30 min introduction of an intruder mouse to the cage and lights off or on. These stressors were paired morning or overnight. All non-stressed groups were given food and water at all times, as well as comfortable cage surroundings, while the experimental group was housed in a different room. Once every week sucrose (2%) consumption was measured as a test of anhedonia in the CMS and non-stressed animals. At the end of the stress regime, alcohol consumption was measured in all groups of the CMS and non-stressed, similar to the anhedonia test regimen.

#### Sucrose consumption test

Sucrose intake (2% sucrose solution) was measured once every week and consumption of sucrose solution was estimated simultaneously in the control and experimental groups by comparing bottle weights before and after the anhedonia test. The volume of sucrose consumed was obtained by the difference before and after the anhedonia test and also by weighing the bottles with the sucrose before and after the anhedonia tests. No difference between the two methods were noted when considering inadvertent spills. The intake was used as the measure of anhedonia – a lack of pleasure validated for the CMS model^63^.

### Statistical analysis

Statistical analysis were performed with Prism version 5.0 (GraphPad Software Inc., La Jolla, CA). The number of studied in each of the behavioral tests was 10 ± 2. Results are expressed as mean ± SEM. Statistical significance was assessed by Mann-Whitney test of Kruskal-Wallis one-way ANOVA test followed by Dunn’s post hoc analysis for multiple comparisons. For the CPP test, data was converted to an initial preference score for each mouse by subtracting the time spent in the drug-paired compartment on day 1 (pre-conditioning phase) from time spent in the same chamber on the last day (post-conditioning phase). Positive scores indicate the development of preference. Behavioral data are presented as mean ± s.e.m. One-way analysis of variance (ANOVA) followed by Bonferroni’s post hoc test for multiple comparisons. Other behavioral analysis were analyzed by the student *t*- test and a *P-value < 0.05 was considered statistically significant.

### Availability of materials and data

The datasets generated during and/or analyzed during the current study are available from the corresponding author on reasonable request^64–69^.

## Electronic supplementary material

Supplementary Information

## References

[1] Onaivi ES (2012). CNS effects of CB2 cannabinoid receptors: beyond neuro-immuno-cannabinoid activity. J. Psychopharmacol.

[2] Marsicano G (2003). CB1 cannabinoid receptors and on-demand defense against excitotoxicity. Science.

[3] Monory K (2006). The endocannabinoid system controls key epileptogenic circuits in the hippocampus. Neuron.

[4] Albayram O (2011). Role of CB1 cannabinoid receptors on GABAergic neurons in brain aging. PNAS.

[5] Zhang HY (2015). Species differences in cannabinoid receptor 2 and receptor responses to cocaine self-administration in mice and rats. Neuropsychopharmacol.

[6] Xi Z-X (2011). Brain cannabinoid CB2 receptors modulate cocaine’s action in mice. Nat. Neurosci.

[7] Stempel AV (2016). Cannabinoid type 2 receptors mediate a cell type-specific plasticity in the hippocampus. Neuron.

[8] Buckley NE (2000). Immunomodulation by cannabinoids is absent in mice deficient for the cannabinoid CB2 receptor. Eur J Pharmacol.

[9] Liu, Q. R. *et al*. Species differences in cannabinoid receptor 2 (CNR2 gene): identification of novel human and rodent CB2 isoforms, differential tissue expression and regulation by cannabinoid ligands. Genes Brain Behav: 519–30 (2009). 10.1111/j.1601-183X.2009.00498.x PMC3389515 19496827

[10] Kendall, D. A. & Yudowski, G. A. Cannabinoid receptors in the central nervous system: Their signaling and roles in disease. *Fron. Cell. Neurosci*. 10:294. 10.3389/fncel.2016.00294. 10.3389/fncel.2016.00294 PMC5209363 28101004

[11] Szczesniak A-M (2017). Cannabinoid 2 receptor is a novel anti-inflammatory target in experimental proliferative vitreoretinopathy. Neuropharmacology.

[12] Cox ML, Haller VL, Welch SP (2007). The antinociceptive effect of Δ^9^-tetrahydrocannabinol in the arthritic rat involves the CB2 cannabinoid receptor. Eur J Pharmacol.

[13] South SM, Smith MT (1998). Apparent insensitivity of the hotplate latency test for detection of antinoception following intraperitoneal, intravenous or intracerebroventricular M6G administration to rats. J Pharmacol Exp Ther.

[14] Soethoudt M (2017). Cannabinoid CB2 receptor ligand profiling reveals biased signaling and off-target activity. Nature Communications.

[15] Onaivi ES (2011). Commentary: Functional neuronal CB2 cannabinoid receptors in the CNS. Current Neuropharmacology.

[16] Onaivi ES (2008). Behavioral effects of CB2 cannabinoid receptor activation and its influence on food and alcohol consumption. Ann. N.Y. Acad. Sci..

[17] Ishiguro H (2007). Involvement of cannabinoid CB2 receptor in alcohol preference in mice and alcoholism humans. The Pharmacogenomics Journal.

[18] Zhang, H.-Y. *et al*. Expression of functional cannabinoid CB2 receptor in VTA dopamine neurons in rats. Addiction Biology 10.1111/adb. 12367 (2016). 10.1111/adb.12367 PMC4969232 26833913

[19] Maia TV, Frank MJ (2011). From reinforcement learning models of the basal ganglia to the pathophysiology of psychiatric and neurological disorders. Nat Neurosci.

[20] Liu, K. *et al*. AZ123′UTR is a new SLC6A3 downregulator associated with an epistatic protection against substance use disorders. *Mol Neurobiol*10.1007/s12035-017-0781-2 [Epub ahead of print] (2017). 10.1007/s12035-017-0781-2 PMC5886844 28983843

[21] den Boon FS (2012). Excitability of prefrontal cortical pyramidal neurons is modulated by activation of intracellular type-2 cannabinoid receptors. PNAS.

[22] Van Sickle MD (2005). Identification and functional characterization of brainstem cannabinoid CB2 receptors. Science.

[23] Onaivi ES (2006). Discovery of the presence and functional expression of cannabinoid CB2 receptors in brain. Ann N Y Acad Sci.

[24] Dotsey E (2017). Transient cannabinoid receptor 2 blockade during immunization heightens intensity and breadth of antigen-specific antibody responses in young and aged mice. Scientific reports.

[25] Pavlopoulos (2006). Cannabinoid receptors as therapeutic targets. Curr.Pharm.Des..

[26] Di Marzo V (2004). The endocannabinoid system and its therapeutic exploitation. Nat. Rev. Drug Discov.

[27] Onaivi, E. S. *et al*. Brain neuronal CB2 cannabinoid receptors in drug abuse and depression: From mice to Human subjects. *PLoS one* 3(2): e16440. 101371 (2008). 10.1371/journal.pone.0001640 PMC2241668 18286196

[28] Sudhof TC, Malenka RC (2008). Understanding synapses: past, present, and future. Neuron.

[29] Katona I, Freund TF (2012). Multiple functions of endocannabinoid signaling in the brain. Ann Rev Neurosci.

[30] Brusco A (2008). Postsynaptic localization of CB2 cannabinoid receptors in the rat hippocampus. Synapse.

[31] Brusco A (2008). Ultrastructural localization of neuronal brain CB2 cannabinoid receptors. Ann N Y Acad Sci.

[32] De Luca MA (2015). Stimulation of *in vivo* dopamine transmission and intravenous self-administration in rats and mice by JWH-018, a spice cannabinoid. Neuropharmacology.

[33] Metna-Laurent M (2017). Cannabinoid-induced tetrad in mice. Current Protocols in Neuroscience.

[34] Wiley JL, Martin BR (2003). Cannabinoid pharmacological properties common to other centrally acting drugs. Eur J Pharmacol.

[35] Atwood BK, Straiker A, Mackie K (2012). CB2 cannabinoid receptors inhibit synaptic transmission when expressed in cultured autaptic neurons. Neuropharmacology.

[36] Morgan NH, Stanford IM, Woodhall GL (2009). Functional CB2 type cannabinoid receptors at CNS synapses. Neuropharmacology.

[37] Ong WY, Mackie K (1999). A light and electron microscopic study of the CB1 cannabinoid receptor in the primate brain. Neuroscience.

[38] Baron, E. P. Comprehensive review of medical marijuana, cannabinoids and therapeutic implications in medicine and headache: What a long strange trip it’s been. *Headache Currents* 885–916 (2015). 10.1111/head.12570 26015168

[39] Svizenska IH (2013). Bilateral changes of cannabinoid receptor type 2 protein and mRNA in the dorsal root ganglia of a rat neuropathic pain model. J Histochem Cytochem.

[40] Hollinshead SP (2013). Selective cannabinoid receptor type 2 (CB2) agonists: Optimization of a series of purines leading to identification of a clinical candidate for the treatment of osteoarthritic pain. J Med Chem.

[41] Yang L (2014). Celastrol attenuates inflammatory and neuropathic pain mediated by cannabinoid receptor type 2. Int J Mol Sci.

[42] Xu J (2016). Activation of cannabinoid receptor 2 attenuates mechanical allodynia and neuroinflammatory responses in a chronic post-ischemic pain model of complex regional pain syndrome type 1 in rats. Euro J Neurosc.

[43] Munro S (1993). Molecular characterization of a peripheral receptor for cannabinoids. Nature.

[44] Griffin G (1999). Evaluation of the cannabinoid CB2 receptor-selective antagonist, SR144528: further evidence for CB2 receptor absence in the rat central nervous system. Eur J Pharmacol..

[45] Onaivi ES (1990). Pharmacological characterization of cannabinoid in the plus maze. The J pharmacol Ther.

[46] Liu Q-R (2014). Detection of molecular alterations in methamphetamine activated Fos-expressing neurons from a single rat dorsal striatum using fluorescence-activated cell sorting (FACS). J. Neurochem.

[47] Aracil-Fernandez A (2012). Decrease cocaine motor sensitization and self-administration in mice overexpressing cannabinoid CB (2) receptors. Neuropsychopharmacology.

[48] Lanciego JL (2011). Expression of the mRNA coding the cannabinoid receptor 2 in the pallidal complex of Macaca fasicularis. J Psychopharmacol.

[49] Eduardo B-C (2014). Pharmacological blockade of either cannabinoid CB1 or CB2 receptors prevents both cocaine-induced conditioned locomotion and cocaine-induced reduction of cell proliferation in the hippocampus of adult male rat. Frontiers in Integrative Neurosci.

[50] Argue, K. J. *et al*. Activation of both CB1 and CB2 endocannabinoid receptors is critical for masculinization of the developing medial amygdala and juvenile social play behavior. *eNeuro* 4(1): e0344-16.2017 1-20 (2017). 10.1523/ENEURO.0344-16.2017 PMC5272923 28144625

[51] Bedse, G. *et al*. Functional redundancy between canonical endocannabinoid signaling systems in the modulation of anxiety. *Biol Psychiat* [ahead of print] (2017). 10.1016/j.biopsych.2017.03.002 PMC5585044 28438413

[52] Quraishi SA, Paladini CA (2016). A central move for CB2 receptors. Neuron.

[53] Morales M, Bonci A (2012). Hooking CB2 receptor into drug abuse?. Nature Medicine.

[54] Rogers N (2015). Cannabinoid receptor with an ‘identity crisis’ gets a second look. Nature Medicine.

[55] Schmole AC (2015). Expression analysis of CB2-GFP BAC transgenic mice. PLoS ONE.

[56] Chen X (2015). Neurotrophic effects of serum- and glucocorticoid-inducible kinase (SGK) on adult murine mesencephalic dopamine neurons. J Neurosci.

[57] Onaivi ES (2011). Consequences of cannabinoid and monoaminergic system disruption in a mouse model of autism spectrum disorders. Curr Neuropharmacol.

[58] Onaivi ES, Martin BR (1989). Neuropharmacological and physiological validation of a computer-controlled two-compartment black and white box for the assessment of anxiety. Prog. Neuro-Psychopharmacol & Biol. Psychiat.

[59] Steru L (1985). The tail suspension test: a new method for screening antidepressants in mice. Psychopharmacol.

[60] Onaivi ES (1990). Pharmacological characterization of cannabinoids in the elevated plus maze. The J Pharmacol Exp Ther.

[61] Fride, E. *et al*. Behavioral methods in cannabinoid research: In Onaivi ES (Ed.) Marijuana and cannabinoid research: Methods and Protocols. Totowa, Humana press Inc, pp 269–290. (2006). 10.1385/1-59259-999-0:269 16506414

[62] Onaivi, E.S. *et al*. Methods to study the behavioral effects and expression of CB2 cannabinoid receptor and its gene transcripts in the chronic mild stress model of depression. In Onaivi ES (Ed.) Marijuana and cannabinoid research: Methods and Protocols. Totowa, Humana press Inc, pp 291–297 (2006). 10.1385/1-59259-999-0:291 16506415

[63] Willner P (2005). Chronic mild stress (CMS) revisited: consistency and behavioral-neurobiological concordance in the effects of CMS. Neuropsychobiology.

[64] Anderson CA (2010). Data quality control in genetic case-control association studies. Nat Protoc.

[65] Kennedy JL (2016). Increased Nigral SLC6A3 Activity in Schizophrenia Patients: Findings from the Toronto-McLean Cohorts. Schizophr Bull..

[66] Purcell S (2007). PLINK: a tool set for whole-genome association and population-based linkage analyses. Am J Hum Genet..

[67] Shang Y, Tang Y (2017). The central cannabinoid receptor type-2 (CB2) and chronic pain. Int J Neurosci.

[68] Xiong N (2016). hVMAT2: A target of individualized medication for Parkinson’s disease. Neurotherapeutics.

[69] Wang X (2016). Genetic variants of microtubule actin cross-linking factor 1 (MACF1) confer risk for Parkinson’s disease. Mol Neurobiol.

