# Supplementary material for: Cannabinoid type 2 receptors in dopamine neurons inhibits psychomotor behaviors, alters anxiety, depression and alcohol preference
Source: Sci Rep. 2017 Dec 12;7:17410. doi: 10.1038/s41598-017-17796-y (PMC5727179; doi:10.1038/s41598-017-17796-y)
Supplement: Supplementary file 1 — Supplementary Information [file 41598_2017_17796_MOESM1_ESM.pdf]

Cannabinoid type 2 receptors in dopamine neurons inhibits psychomotor behaviors, alters anxiety, depression and alcohol preference

Qing-Rong Liu[1,2], Ana Canseco-Alba[1], Hai-Ying Zhang[3], Patricia Tagliaferro[1], Monika Chung[1], Eugene Dennis[1], Branden Sanabria[1], Norman Schanz[1], Joao Carlos Escosteguy-Neto[1], Hiroki Ishiguro[4], Zhicheng Lin[5], Susan Sgro[1], Claire M. Leonard[1], Jair Guilherme Santos-Junior[6], Eliot L. Gardner[3], Josephine M. Egan[2], Jeung Woon Lee[1], Zheng-Xiong Xi[3], & Emmanuel S. Onaivi[1]

SUPPLEMENTARY METHODS AND DATA

Human CNR2 GWAS analysis

The GWAS secondary analysis used dbGaP three datasets, Collaborative Study on the Genetics of Alcoholism (COGA, phs000125.v1.p1), Study of Addiction: Genetics and Environment (SAGE, phs000092.v1.p1) and the Australian twin-family study of alcohol use disorder (OZALC, phs000181.v1.p1). COGA had two ethnicities US Caucasians and African American so after data quality control (QC) [60], the dataset was split into two cohorts, US Caucasians and African Americans. Only the unrelated individuals were extracted from the OZALC sample for the purpose of epistasis analysis. After QC, a total of 6,596 unrelated subjects remained, including approximately 41.0% cases. Imputation was carried out as described before [61], in order to extend the association power. Data manipulation, allelic association and meta-analysis were all performed by using PLINK [62] and meta-analysis of the three dbGaP GWAS datasets for CNR2 association with Parkinson's disease as previously determined [64, 65].

SUPPLEMENTARY FIGURE S1. Human CNR2 gene association with SUD, based on dbGaP datasets. Human CNR2 gene association with SUD, based on dbGaP datasets. Data are

results from meta-analysis of three GWAS studies, including COGA, SAGE and the Australian Twins study, together with a total of 6,596 subjects. X axis is coordinate in chr1, Y axis is significance of association in the form of $-\text{Log}_{10}$(P-value). Blue line is nominal significance of P $\leq$ 0.05. Aquamarine, CNR2 gene structure corresponding to chr1, running in the minus strand of the chromosome. Red arrow, the most significant association via SNP rs3123557.

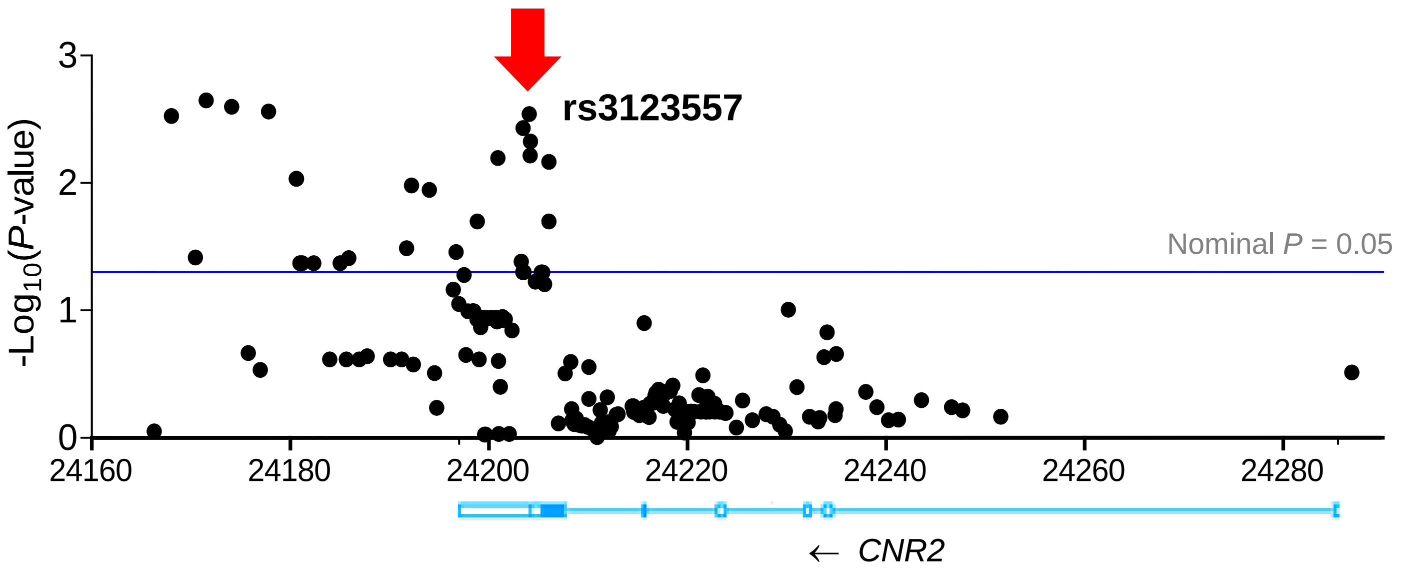

Table S1. Results of meta-analysis of three of dbGaP GWAS datasets for CNR2 association with PD.

CHR	BP	SNP	A1	A2	N	P(R)	OR(R)	
1	24153608	rs7521571	G	A	2	0.02003	0.9124	
1	24166191	rs12123991	A	G	2	0.6659	0.979	
1	24168019	rs12744386	T	C	3	0.3578	0.9682	
1	24171543	rs4649119	G	C	3	0.3752	0.9693	
1	24174093	rs10917431	G	T	3	0.3669	0.9688	
1	24177800	rs72874118	G	A	3	0.3477	0.9675	
1	24192200	rs34902309	G	A	3	0.3478	0.9675	
1	24207691	rs4474201	G	T	2	0.02175	0.9132	3'UTR
1	24219690	rs7553231	T	C	2	0.9796	0.9987	
1	24230163	rs6700106	A	G	2	0.5567	0.9695	

Table S2. Results of meta-analysis of three of dbGaP GWAS datasets for CNR2 association with SUD.

CHR	BP	SNP	A1	A2	N	P	P(R)	OR	OR(R)
1	24166289	rs187512781	C	G	2	0.891	0.891	0.9752	0.9752
1	24168019	rs12744386	T	C	3	0.002977	0.002977	1.1213	1.1213
1	24170458	rs10917430	T	C	2	0.03828	0.03828	1.1087	1.1087
1	24171543	rs4649119	G	C	3	0.002242	0.002242	1.1249	1.1249
1	24174093	rs10917431	G	T	3	0.002522	0.002522	1.1233	1.1233
1	24175763	rs12404321	G	A	2	0.2155	0.2155	1.1016	1.1016
1	24176983	rs77767474	A	T	2	0.2924	0.2924	1.0853	1.0853
1	24177800	rs72874118	G	A	3	0.002749	0.002749	1.1227	1.1227
1	24180613	rs35993651	G	C	2	0.009269	0.009269	1.1303	1.1303
1	24180623	rs35904775	C	T	2	0.009269	0.009269	1.1303	1.1303
1	24180962	rs13551	C	T	2	0.04256	0.04256	1.1062	1.1062
1	24181177	rs12748109	T	C	2	0.04256	0.04256	1.1062	1.1062
1	24182348	rs11803575	T	A	2	0.04256	0.04256	1.1062	1.1062
1	24183981	rs74223775	A	G	2	0.2422	0.2422	1.0945	1.0945
1	24185025	rs12755062	A	T	2	0.04256	0.04256	1.1062	1.1062
1	24185648	rs78199675	C	A	2	0.2422	0.2422	1.0945	1.0945
1	24185877	rs12141409	T	C	2	0.03876	0.03876	1.1085	1.1085
1	24186934	rs145447703	C	T	2	0.2422	0.2422	1.0945	1.0945
1	24187738	rs140967752	A	G	2	0.229	0.229	1.0974	1.0974
1	24190106	rs143150363	G	C	2	0.2422	0.2422	1.0945	1.0945
1	24191219	rs12133557	T	G	2	0.2422	0.2422	1.0945	1.0945
1	24191720	rs34570472	G	A	3	0.03247	0.03247	1.1048	1.1048
1	24192200	rs34902309	G	A	4	0.007754	0.01046	1.1023	1.1012
1	24192379	rs74937660	C	G	2	0.2659	0.2659	1.0904	1.0904
1	24194014	rs3003333	T	A	2	0.01134	0.01134	1.1267	1.1267
1	24194522	rs4317761	G	T	3	0.3105	0.3105	1.0441	1.0441
1	24194748	rs2070956	G	C	3	0.5798	0.5798	1.0394	1.0394
1	24196401	rs3123554	A	G	3	0.0197	0.06841	1.1073	1.0992
1	24196683	rs4285654	C	T	3	0.01782	0.03485	1.108	1.1047
1	24196957	rs2503002	C	G	3	0.0208	0.08911	1.1063	1.0962
1	24197512	rs2503001	G	A	3	0.02284	0.05278	1.1047	1.0995
1	24197696	rs4649123	A	G	3	0.02621	0.2236	1.1025	1.0815
1	24197898	rs2503000	C	G	3	0.02142	0.1012	1.1057	1.0945
1	24198321	rs2502999	A	G	3	0.02142	0.1012	1.1057	1.0945
1	24198327	rs2502998	T	A	3	0.02142	0.1012	1.1057	1.0945
1	24198441	rs2502997	G	A	3	0.02391	0.1012	1.1038	1.0933
1	24198479	rs2501417	T	C	3	0.02142	0.1012	1.1057	1.0945
1	24198804	rs6680132	T	C	3	0.02363	0.1174	1.1039	1.0916
1	24198840	rs6672157	C	G	3	0.01636	0.01997	1.1107	1.1098

1	24199026	rs2501423	A	C	3	0.02697	0.2425	1.102	1.0797
1	24199041	rs2502996	T	C	3	0.02211	0.1145	1.1051	1.0927
1	24199182	rs2501424	T	C	3	0.03121	0.135	1.0971	1.0858
1	24199290	rs2502995	T	C	3	0.02211	0.1145	1.1051	1.0927
1	24199339	rs2501425	A	C	3	0.02205	0.1136	1.1052	1.0929
1	24199576	rs74063322	C	T	2	0.9412	0.9412	0.987	0.987
1	24199642	rs6663474	G	A	3	0.02211	0.1145	1.1051	1.0927
1	24199665	rs74063324	A	G	2	0.9406	0.9406	0.9868	0.9868
1	24199869	rs3003334	G	A	3	0.02211	0.1145	1.1051	1.0927
1	24200052	rs3003335	A	G	3	0.02211	0.1145	1.1051	1.0927
1	24200158	rs6665733	T	C	3	0.02211	0.1145	1.1051	1.0927
1	24200524	rs1130321	C	T	3	0.02211	0.1145	1.1051	1.0927
1	24200606	rs1130320	A	G	3	0.02211	0.1145	1.1051	1.0927
1	24200772	rs1106	C	G	3	0.02211	0.1145	1.1051	1.0927
1	24200800	rs1105	T	C	3	0.03198	0.122	1.0982	1.0876
1	24200903	rs2229586	G	C	2	0.006364	0.006364	1.1369	1.1369
1	24200921	rs2229585	T	C	3	0.02405	0.1183	1.1036	1.0913
1	24200969	rs2229584	T	C	3	0.02405	0.1183	1.1036	1.0913
1	24200983	rs2229583	C	T	3	0.02928	0.2493	1.1005	1.0782
1	24201002	rs2229582	C	G	2	0.9282	0.9282	0.9841	0.9841
1	24201094	rs2229581	G	C	3	0.02405	0.1183	1.1036	1.0913
1	24201109	rs2229580	C	T	3	0.02405	0.1183	1.1036	1.0913
1	24201162	rs2229579	A	G	3	0.3964	0.3964	1.066	1.066
1	24201262	rs2502993	A	G	3	0.02363	0.1174	1.1039	1.0916
1	24201357	rs4649124	A	G	3	0.02105	0.1126	1.106	1.0936
1	24201448	rs3003336	C	T	3	0.02405	0.1183	1.1036	1.0913
1	24201643	rs2501431	G	A	3	0.02363	0.1174	1.1039	1.0916
1	24202055	rs2506088	G	A	3	0.9292	0.9292	1.0069	1.0069
1	24202318	rs2502991	A	G	3	0.01835	0.143	1.109	1.0923
1	24203246	rs2501433	A	G	3	0.01382	0.04132	1.1142	1.1079
1	24203416	rs6658703	G	T	3	0.0143	0.05005	1.1136	1.106
1	24203425	rs2501434	C	T	2	0.003698	0.003698	1.1461	1.1461
1	24203534	rs6673210	A	G	3	0.0143	0.05005	1.1136	1.106
1	24204073	**rs3123557**	C	T	2	0.002879	0.002879	1.1513	1.1513
1	24204143	rs6424119	A	G	2	0.006094	0.006094	1.1381	1.1381
1	24204189	rs6424120	C	T	2	0.004727	0.004727	1.1423	1.1423
1	24204683	rs4341315	C	G	3	0.01475	0.05942	1.113	1.1043
1	24205274	rs2501367	C	A	3	0.01429	0.0503	1.1136	1.106
1	24205295	rs2502990	C	T	3	0.01429	0.0503	1.1136	1.106
1	24205407	rs2502989	G	A	3	0.01429	0.0503	1.1136	1.106
1	24205608	rs2502988	T	C	3	0.02007	0.06216	1.1078	1.1002
1	24206032	rs2501369	G	A	3	0.01745	0.02	1.1102	1.1095
1	24206053	rs2501370	T	C	2	0.006812	0.006812	1.1368	1.1368
1	24206985	rs6689530	T	C	3	0.7912	0.7693	0.9844	0.9817

1	24207691	rs4474201	G	T	4	0.3109	0.3109	1.0372	1.0372
1	24208236	rs12727568	T	C	2	0.2529	0.2529	0.9096	0.9096
1	24208320	rs76048179	T	G	3	0.5927	0.5927	0.9621	0.9621
1	24208373	rs12754324	C	T	3	0.7308	0.7308	1.0172	1.0172
1	24208574	rs2506086	T	G	3	0.7806	0.7806	0.9787	0.9787
1	24208676	rs2501377	T	C	3	0.7806	0.7806	0.9787	0.9787
1	24208783	rs7541711	C	T	3	0.7042	0.7042	1.0189	1.0189
1	24208789	rs7541713	C	A	3	0.7042	0.7042	1.0189	1.0189
1	24208875	rs7541819	C	T	3	0.778	0.778	1.014	1.014
1	24208885	rs7532916	G	T	3	0.778	0.778	1.014	1.014
1	24208924	rs114636165	C	T	3	0.7602	0.7602	0.9492	0.9492
1	24208934	rs7541841	C	T	3	0.778	0.778	1.014	1.014
1	24209034	rs12741866	A	G	3	0.786	0.786	1.0135	1.0135
1	24209168	rs12759455	C	A	3	0.7937	0.7937	1.013	1.013
1	24209378	rs12733278	T	C	3	0.8016	0.8016	1.0125	1.0125
1	24209630	rs12759917	G	T	3	0.7937	0.7937	1.013	1.013
1	24209971	rs4625225	C	T	3	0.8207	0.8207	1.0112	1.0112
1	24210067	rs7528292	C	T	3	0.278	0.278	0.9272	0.9272
1	24210080	rs75296863	T	C	3	0.4947	0.4947	1.0469	1.0469
1	24210453	rs7519729	G	C	3	0.8552	0.8552	1.009	1.009
1	24210894	rs61778195	C	A	2	0.9877	0.9877	0.9991	0.9991
1	24211206	rs76135131	A	C	3	0.6031	0.6031	1.0335	1.0335
1	24211342	rs4535949	G	C	3	0.7661	0.7661	1.0151	1.0151
1	24211913	rs3923183	T	C	3	0.4804	0.4804	0.9501	0.9501
1	24212044	rs4501783	G	T	3	0.8814	0.8814	1.0074	1.0074
1	24212100	rs5026902	C	T	3	0.88	0.88	1.0076	1.0076
1	24212106	rs183017791	C	A	3	0.7512	0.7512	0.9473	0.9473
1	24212336	rs4648919	G	C	3	0.8137	0.8137	1.0118	1.0118
1	24212829	rs6424123	C	T	3	0.6598	0.6598	1.0223	1.0223
1	24212999	rs7537808	C	T	3	0.6494	0.6494	1.023	1.023
1	24214430	rs2501385	C	T	3	0.5635	0.5635	1.0348	1.0348
1	24214550	rs2502956	T	C	3	0.5635	0.5635	1.0348	1.0348
1	24214583	rs2501386	A	G	3	0.6252	0.6252	1.0294	1.0294
1	24214721	rs2501387	A	G	3	0.6228	0.6228	1.0293	1.0293
1	24214868	rs2501388	A	G	3	0.63	0.63	1.0287	1.0287
1	24215130	rs16828926	A	G	3	0.6612	0.6612	1.0278	1.0278
1	24215473	rs61778196	T	G	3	0.6206	0.6206	1.0294	1.0294
1	24215477	rs6699263	T	G	3	0.6206	0.6206	1.0294	1.0294
1	24215574	rs3003322	T	C	3	0.6086	0.6086	1.0305	1.0305
1	24215592	rs6682096	G	A	3	0.5803	0.5803	1.0329	1.0329
1	24215595	rs6691618	C	T	3	0.6143	0.6143	1.03	1.03
1	24215618	rs2501389	C	G	3	0.6086	0.6086	1.0305	1.0305
1	24215652	rs6696395	A	C	2	0.1256	0.1256	0.7439	0.7439
1	24215662	rs6424125	T	G	3	0.6576	0.6576	1.026	1.026

1	24215749	rs7554234	T	C	3	0.6086	0.6086	1.0305	1.0305
1	24215796	rs2501390	A	G	3	0.6086	0.6086	1.0305	1.0305
1	24216118	rs2501391	G	A	3	0.6871	0.6871	1.0235	1.0235
1	24216181	rs2501392	G	C	3	0.5421	0.5421	1.0363	1.0363
1	24216393	rs2501393	T	G	3	0.5282	0.5282	1.0379	1.0379
1	24216541	rs2502957	T	C	3	0.5282	0.5282	1.0379	1.0379
1	24216723	rs2502958	C	T	3	0.4687	0.4687	1.0439	1.0439
1	24216811	rs2501395	T	C	3	0.4417	0.4417	1.0466	1.0466
1	24216861	rs2502959	A	C	3	0.449	0.449	1.0458	1.0458
1	24217088	rs6424126	A	G	2	0.4174	0.4174	1.0586	1.0586
1	24217092	rs7541728	G	A	3	0.4639	0.4639	1.0438	1.0438
1	24217513	rs2502960	C	T	3	0.4518	0.4518	1.0455	1.0455
1	24217567	rs2502961	A	G	3	0.5608	0.5608	1.0351	1.0351
1	24217620	rs2501396	T	A	3	0.4518	0.4518	1.0455	1.0455
1	24217692	rs2501397	C	T	3	0.4518	0.4518	1.0455	1.0455
1	24218064	rs2502964	A	G	3	0.4283	0.4283	1.048	1.048
1	24218276	rs2502965	G	A	3	0.4283	0.4283	1.048	1.048
1	24218530	rs4285653	C	T	3	0.3875	0.3875	0.9546	0.9546
1	24218735	rs3003323	T	G	3	0.5961	0.5961	0.9718	0.9718
1	24218742	rs4573477	G	A	3	0.5961	0.5961	0.9718	0.9718
1	24218814	rs3003324	T	C	3	0.5961	0.5961	0.9718	0.9718
1	24218960	rs2502966	G	A	2	0.3189	0.7479	0.8322	1.2782
1	24219158	rs2501398	G	T	2	0.5365	0.5365	0.9642	0.9642
1	24219234	rs6424127	T	C	3	0.6143	0.6143	0.9732	0.9732
1	24219242	rs6424128	C	T	3	0.6076	0.6076	0.9727	0.9727
1	24219293	rs6424129	G	A	3	0.6004	0.6004	0.9721	0.9721
1	24219374	rs6424130	T	C	3	0.5998	0.5998	0.9721	0.9721
1	24219582	rs7512349	C	T	3	0.6205	0.6205	0.9736	0.9736
1	24219636	rs7512373	C	T	2	0.2575	0.6854	0.8094	0.868
1	24219690	rs7553231	T	C	4	0.911	0.911	0.9952	0.9952
1	24219717	rs2502967	A	G	3	0.6205	0.6205	0.9736	0.9736
1	24219758	rs2501399	C	A	3	0.6205	0.6205	0.9736	0.9736
1	24219800	rs2501400	C	T	3	0.6205	0.6205	0.9736	0.9736
1	24219849	rs2502968	A	G	3	0.6205	0.6205	0.9736	0.9736
1	24220063	rs2501401	A	G	3	0.7568	0.7568	0.9835	0.9835
1	24220278	rs2502969	T	C	3	0.6205	0.6205	0.9736	0.9736
1	24220343	rs2502970	A	G	3	0.6205	0.6205	0.9736	0.9736
1	24220407	rs2501402	G	A	3	0.6205	0.6205	0.9736	0.9736
1	24220606	rs6424131	C	A	3	0.6205	0.6205	0.9736	0.9736
1	24221135	rs12730734	T	A	3	0.4609	0.4609	0.9608	0.9608
1	24221321	rs2501403	T	A	3	0.6205	0.6205	0.9736	0.9736
1	24221539	rs28735813	G	A	3	0.3225	0.3225	0.9459	0.9459
1	24221817	rs9424397	G	T	3	0.6205	0.6205	0.9736	0.9736
1	24221834	rs9424398	T	G	3	0.6205	0.6205	0.9736	0.9736

1	24221918	rs12724034	C	A	3	0.6205	0.6205	0.9736	0.9736
1	24221935	rs9424399	G	A	2	0.5389	0.5389	0.9645	0.9645
1	24222029	rs2502971	G	A	2	0.4749	0.4749	0.9587	0.9587
1	24222197	rs9424400	A	G	3	0.6205	0.6205	0.9736	0.9736
1	24222735	rs9424338	G	T	2	0.5389	0.5389	0.9645	0.9645
1	24222771	rs2501404	G	A	3	0.6205	0.6205	0.9736	0.9736
1	24223226	rs3003327	G	A	3	0.6205	0.6205	0.9736	0.9736
1	24223245	rs3003623	G	A	3	0.6205	0.6205	0.9736	0.9736
1	24223775	rs2502973	A	T	3	0.637	0.637	0.9748	0.9748
1	24223859	rs2502974	T	G	3	0.637	0.637	0.9748	0.9748
1	24224933	rs11488707	A	G	2	0.8305	0.8305	0.9371	0.9371
1	24225530	rs2501406	T	C	2	0.4744	0.5094	1.127	1.191
1	24226528	rs61778212	G	A	3	0.7258	0.7258	0.9795	0.9795
1	24227934	rs61778213	T	C	3	0.6499	0.6499	0.9738	0.9738
1	24228618	rs6669556	C	T	2	0.6807	0.6807	0.9757	0.9757
1	24229276	rs6680554	T	C	2	0.8665	0.786	1.0892	1.2237
1	24229825	rs75761020	A	G	2	0.885	0.885	1.0793	1.0793
1	24230163	rs6700106	A	G	4	0.09845	0.09845	0.9263	0.9263
1	24231016	rs4259582	A	G	3	0.4004	0.4004	0.9177	0.9177
1	24232289	rs2501409	C	A	3	0.6811	0.6811	0.9581	0.9581
1	24233151	rs28695556	T	C	2	0.7426	0.7426	0.9752	0.9752
1	24233312	rs6658495	G	A	3	0.6987	0.6987	0.9603	0.9603
1	24233748	rs2501411	G	T	2	0.2321	0.2321	1.8035	1.8035
1	24234041	rs9728437	C	T	2	0.1482	0.1482	2.1193	2.1193
1	24234853	rs2501412	C	T	3	0.6618	0.6618	0.9566	0.9566
1	24234958	rs2501413	C	A	3	0.5927	0.5927	0.9474	0.9474
1	24234965	rs2501414	C	A	2	0.2194	0.2194	2.022	2.022
1	24237962	rs9424339	A	T	2	0.436	0.436	0.9523	0.9523
1	24239078	rs2501415	T	G	2	0.5904	0.5723	1.0807	1.3585
1	24240233	rs114720170	G	T	2	0.74	0.7248	0.9413	0.9345
1	24241216	rs4649138	G	A	3	0.7177	0.7177	1.0386	1.0386
1	24243574	rs2502977	T	C	3	0.5658	0.5042	1.067	1.1567
1	24246588	rs2501420	G	A	3	0.5734	0.5734	1.0598	1.0598
1	24247687	rs9330297	C	A	3	0.6092	0.6092	1.0541	1.0541
1	24251543	rs7537933	G	A	3	0.6804	0.6804	1.0434	1.0434
1	24286897	rs17338135	T	C	3	0.3063	0.3063	1.0445	1.0445
							0.05		

Western blotting

Midbrain region containing the PAG and substantia nigra of C57BL 6/J and DAT-Cnr2 (male, 3 month old) were homogenized mechanically in RIPA buffer (Santa Cruz Biotechnology) and centrifuged for 15 min at 12,000 RPM to collect supernatant. The level of tyrosine hydroxylase (TH) was measured by immunoprecipitation. Briefly, samples were mixed with an equal volume of Laemmli buffer, heated at 100 C for 5 min and separated on precast 4-20% SDS-polyacrylamide gels (Bio-Rad, Hercules, CA). The gel was activated for total protein quantitation and transferred to a PVDF membrane using Trans-Blot Turbo system (Bio-Rad, Hercules, CA). The membrane was placed in blocking solution (TBS with 1% Casein) for 1hr and incubated in anti-TH primary antibody (1:2000, Santa Cruz Biotechnology) overnight at room temperature. Membrane was washed in TBS, incubated in anti-rabbit secondary antibody (1:2000, Bio-Rad) for 1hr and visualized using ECL chemiluminescence solution (Bio-Rad). Image was acquired using the ChemiDoc XRS+ system and Image Lab software.

SUPPLEMENTARY FIGURE S2. Representative Western blot image of TH in the midbrain of naïve C57Bl6/J and DAT-*Cnr2* cKO mice. The TH expression was observed in undiluted (1:1) and one-half dilution (1:2) protein loads, showing the expression of TH in the midbrain of DAT-*Cnr2* cKO mice when compared to the WT mice.

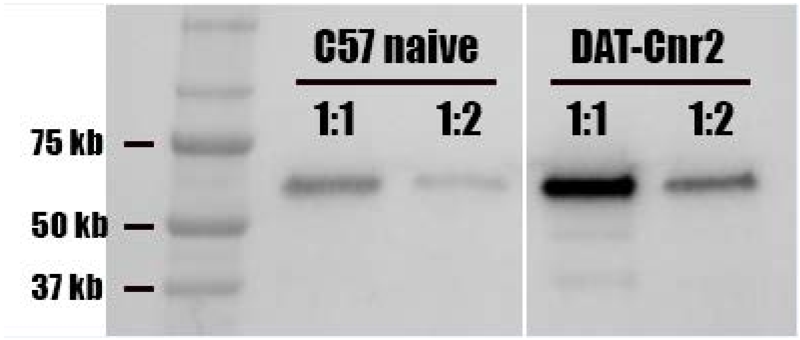

Table S3. Primers for *Cnr2*-flox and DAT-*Cre* transgenic mice

**Transgenic mouse**	**Primer name**	**Primer sequence**
Cnr2-flox	NDEL1	GGTCAAGAATTATGATGCCCTAAGGACC
	NDEL2	CCCAACTCCTTCTGCTTATCCTTCAGG
DAT-*Cre*	Com F	TGGCTGTTGGTGTAAAGTGG
	DAT-WT-F	GGACAGGGACATGGTTGACT
	DAT-KO -R	CCAAAAGACGGCAATATGGT
